# Broad-spectrum vaccines against various and evolving viruses: from antigen design to nanoparticle delivery

**DOI:** 10.1128/jvi.00997-25

**Published:** 2025-09-12

**Authors:** Mengxiang Cao, Yongfeng Li, Xin Song, Zhanhao Lu, Huanjie Zhai, Hua-Ji Qiu, Yuan Sun

**Affiliations:** 1 State Key Laboratory for Animal Disease Control and Prevention, Harbin Veterinary Research Institute, Chinese Academy of Agricultural Sciences 687216 , Harbin, China; Universiteit Gent, Merelbeke, Belgium

**Keywords:** broad-spectrum immune response, vaccine, antigen design, nanoparticle delivery

## Abstract

Pathogen evolution and narrow vaccine coverage urgently demand broad-spectrum vaccines. This review explores two pivotal technological fronts: structural biology- and immunoinformatics-guided antigen design, and utilizing nanoparticle-based delivery systems to induce broad immune responses. We critically analyze four antigen optimization strategies: (i) structure-based antigen design, (ii) conserved epitope targeting, (iii) consensus sequence-based antigen engineering, and (iv) chimeric immunogen design. Additionally, the common types and characteristics of nanoparticles are described briefly. Subsequently, we delve into cutting-edge applications of nanoparticles to enhance immune protection, including mosaic and cocktail nanoparticle vaccines, surface-modified targeting strategies, and the integration of mRNA technology with virus-like particles (VLPs). In conclusion, this review synthesizes risk-benefit analyses of existing strategies, current challenges, and emerging opportunities, offering practical frameworks to facilitate broad-spectrum vaccine innovation and enhance pandemic preparedness.

## INTRODUCTION

**Fig 1:**
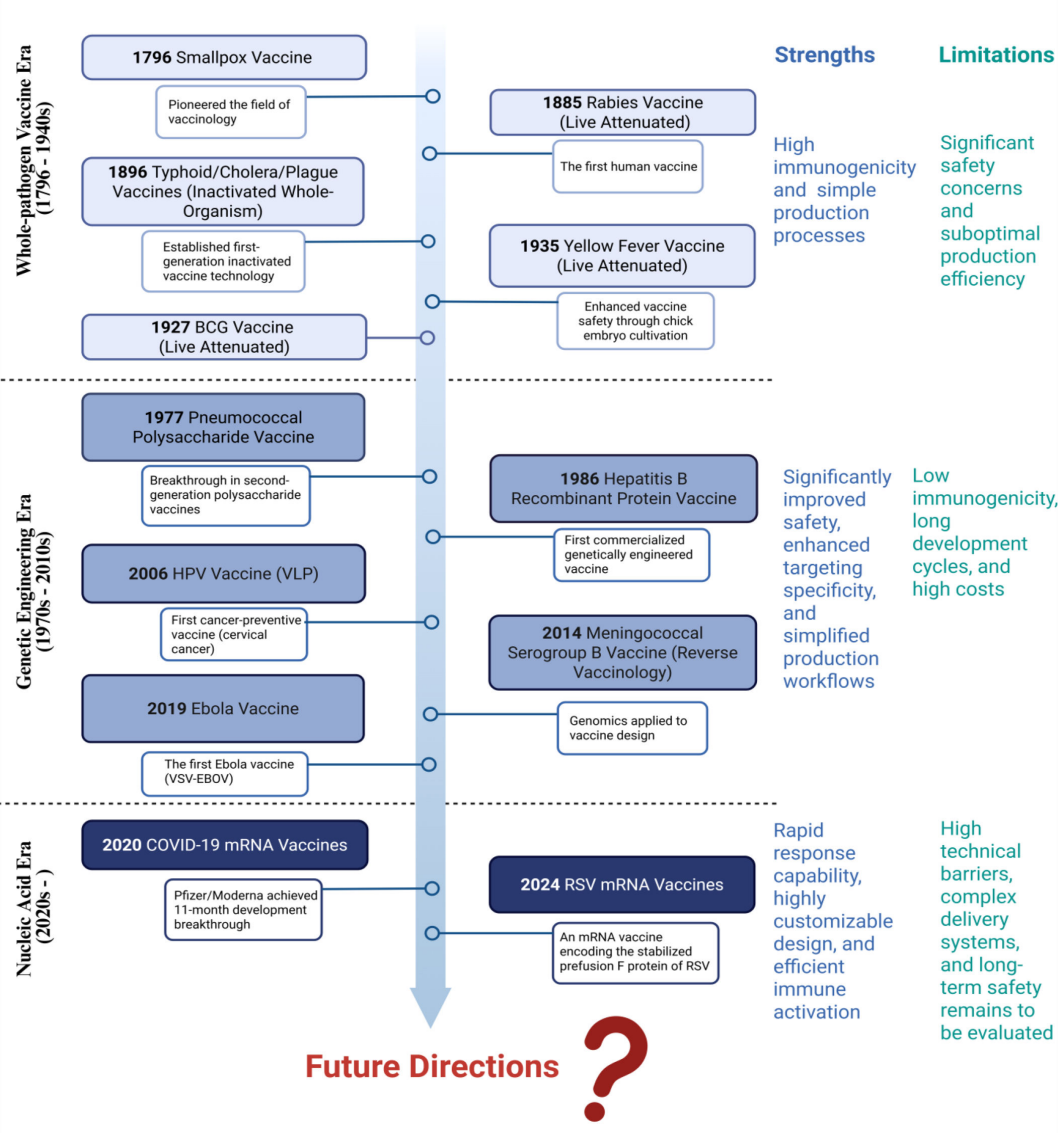
Milestones in human vaccine development. This timeline highlights key advancements in vaccinology. The whole-pathogen vaccine era (1796–1940s) featured foundational vaccines like the smallpox, rabies, and typhoid/cholera/plague vaccines. It offered high immunogenicity but safety risks. The genetic engineering era (1970s–2010s) includes polysaccharide/recombinant protein vaccines (pneumococcal/hepatitis B), VLP-based HPV (first cancer vaccine), reverse vaccinology (meningococcal B), and viral vectors (Ebola vaccine), prioritizing safety and enhanced targeting yet facing immunogenicity challenges. The nucleic acid era (2020s–now) is exemplified by COVID-19 and RSV mRNA vaccines, representing a significant leap in vaccine technology with the application of genomics and the mRNA platform. It offers rapid and flexible design but has high technical barriers and long-term safety yet to be evaluated.

Vaccines serve as powerful tools against infectious diseases. The evolution of vaccine technologies has progressed through three transformative eras (Fig. 1). The first-generation vaccines, like Jenner’s smallpox vaccine and Pasteur’s rabies vaccine, were developed using empirical methods based on inactivated or live-attenuated pathogens. However, these vaccines had significant limitations regarding safety concerns and manufacturing efficiency. The molecular biology revolution in the 1980s enabled second-generation vaccines based on precise antigen-targeting strategies, which significantly enhanced vaccine safety profiles while streamlining manufacturing processes through targeted antigen production and genome-guided immunogen design. The COVID-19 pandemic has unprecedentedly accelerated breakthroughs of third-generation vaccines, with mRNA vaccines achieving a paradigm shift through rapid design and *in vivo* protein expression.

However, the efficacy of vaccines is often limited by a fundamental challenge: antigenic escape driven by pathogen evolution, such as in influenza viruses and SARS-CoV-2 (1, 2). Following the 1918 Spanish flu pandemic (estimated fatalities exceeding 51 million), five waves of influenza pandemic have occurred. China’s current influenza vaccines, trivalent (IIV3) and quadrivalent (IIV4) inactivated vaccines, along with live-attenuated nasal spray vaccine (LAIV3), provide 40% to 60% efficacy against matched strains but lack broad cross-reactivity. Consequently, annual updates are required due to antigenic drift and shift. However, vaccine production faces high costs, delays, and risks of strain mismatch from viral evolution during manufacturing cycles. Similarly, SARS-CoV-2 exemplifies rapid pathogen evolution. It spreads globally at an estimated rate of (8–9) × 10^−4^ nucleotide substitutions per site per year (3), evolving through mutation and recombination to generate variants with enhanced transmissibility, pathogenicity, and immune evasion capabilities (4). This relentless evolutionary pressure underscores the limitations of the traditional, passive vaccine model—the perpetual “chasing the virus” approach. Confronted with this “evolutionary arms race”, this reactive approach has shown limitations, necessitating a paradigm shift from a “reactive response to current strains” to “proactive prevention of future threats”. To counter viral evolution and antigenic diversity, broad-spectrum vaccines targeting conserved epitopes across variants or entire virus families are urgently needed, surpassing traditional strain-specific approaches. Next-generation strategies must integrate two pillars: (1) rational antigen design targeting conserved regions and (2) engineered delivery systems optimized for immune potentiation. The rational design of antigen structures can enhance immune responses in magnitude and breadth (5). Nanoparticles serve as modular delivery platforms. They enhance antigen immunogenicity through multivalent display, lymph node targeting, and controlled release kinetics (6). Combining antigen design with nanoparticle delivery, these strategies collectively overcome traditional vaccine limitations and enable more effective broad-spectrum solutions.

This article primarily focuses on antigen design and nanoparticle delivery systems as innovative approaches to induce broad immune protection. We will first elaborate on various strategies for antigen design, followed by an introduction to the diverse types of nanoparticles and discuss their applications in enhancing immune protection. The review concludes with an evaluation of the strengths and limitations of these strategies, aiming to propose a synergistic paradigm that can effectively address viral diversity and advance pandemic preparedness.

## ANTIGEN DESIGN STRATEGIES TO ENHANCE THE MAGNITUDE AND BREADTH OF IMMUNE PROTECTION

The evolutionary arms race between pathogens and the host immune system poses a fundamental challenge to vaccine development, particularly for rapidly mutating viruses and antigenically diverse pathogens. To overcome immune escape mechanisms and elicit robust and durable protection, next-generation antigen design has evolved beyond empirical approaches toward rational engineering frameworks. In this section, we summarize the innovative antigen design strategies outlined in Table 1, analyzing their respective strengths and limitations.

**TABLE 1:** Analysis of different antigen design strategies

Strategy	Principle	Targeted pathogen/antigen	Core technical methods	Advantages	Challenges/limitations	Stages	References
Structural stabilization	Introducing disulfide bonds, salt bridges, hydrophobic residues, removing buried charged residues, employing prolines	HIV-1 Env trimer	SOSIP	Enhancing trimer solubility and mimicking native Env structure	Limiting applicability, low yield, and instability	Pre-clinical trials	(7)
			NFL2P	Improving yield and stability	Constraining broad applicability	Pre-clinical trials	(8)
			UFO	Enhancing universal applicability and stability	Complex purification methods	Pre-clinical trials	(9)
		The F proteins of RSV, hMPV, HeV, NiV, and hPIV	Introducing disulfide bonds, cavity-filling mutations, and charge neutralization mutation	Enhancing the conformational stability, immunogenicity, and durable and potent neutralizing antibody activity	Th2-skewed immune polarization risk of RSV; multistep purification complexity	Approved (GSK/Pfizer/Moderna)	(10–15)
		SARS-CoV-2 S protein	2P 6P	Improving yield, stability, and eliciting more durable and potent immune response	Need to frequent updates	Approved (Moderna/BioNTech/Johnson & Johnson/Novavax)	(16–19)
		Ebola GP protein	Removing the MPER and TM, fusing the “foldon” domain of T4 fibritin, and introducing a T42A mutation	Enhancing the conformational stability and eliciting more durable and potent immune response	Lacking the MPER region in GP may affect the antibody response	Pre-clinical trials	(20)
Epitope-focused design	Focusing on conserved viral epitopes	SARS-CoV-2, RSV, IAV, and ASFV	Introducing epitope masking, epitope scaffolding, protein dissection, antigen resurfacing, and cross-strain boosting	Steering immune recognition toward defined antigenic regions, thereby broadening protective efficacy	Weakened immunogenicity	Pre-clinical trials	(21–26)
Consensus sequence design	Generating conserved residues encoding cross-reactive epitopes to elicit broad immunity	Dengue virus, SARS-CoV-2, Chikungunya virus, NiV, and influenza virus	Computationally optimized broadly reactive antigens	Covering multiple strains/subtypes to mitigate the impact of antigenic drift	Susceptibility to high mutation rates	Pre-clinical trials	(27–32)
Chimeric antigen design	Integrating key antigenic epitopes from different viral strains or variants into a single immunogen	HuNoV and SARS-CoV-2	Epitope transplantation	Eliciting cross-reactive immune responses against multiple variants	Limited immunogenicity	Pre-clinical trials	(33–35)
		SARS-CoV-2 and influenza virus	Domain substitution	Conferring broad protection across variants/pathogens	Addressing stability limitations and epitope immunodominance hierarchies requires precise engineering to balance antigenic responses	Pre-clinical trials	(36–39)
		Influenza virus and RABV	Incorporating immunomodulatory molecules such as CXCL13, CCL3, XCL1, or GM-CSF	Enhancing non-specific immune responses	Low antigen expression and solubility	Pre-clinical trials	(40–44)

### Antigenic structure stabilization: locking viral proteins in prefusion conformation

Viral surface proteins often undergo conformational changes post-infection, masking critical neutralizing epitopes. Stabilizing these antigens in prefusion states is thus essential for eliciting potent and durable immunity. This section focuses on three major viruses, showcasing how structural vaccinology advances enable prefusion-stabilized vaccine design.

Human immunodeficiency virus (HIV) vaccine development faces challenges due to the conformational flexibility and glycan shield of Env trimers, which hinders the induction of broadly neutralizing antibody (bNAb). To overcome these challenges, three landmark engineering strategies have been developed: SOSIP (the stabilized outer surface immunogenic polypeptide), NFL2P (native flexible linked 2P), and UFO (uncleaved prefusion-optimized design). The SOSIP approach employs multiple modifications to stabilize the Env trimers. These include introducing a disulfide bond and an I559P substitution, removing the hydrophobic membrane-proximal external region (MPER), and mutating the gp120–gp41 cleavage site from REKR to RRRRRR (7). These changes improve the trimers’ solubility, mimic native Env structures, and facilitate bNAb binding while minimizing non-NAb interference. Despite these advances, SOSIP trimers show limitations in applicability, yield, and stability. The NFL2P strategy builds upon SOSIP by eliminating the furin cleavage site and inserting two flexible (G_4_S)_2_ linkers between gp120 and gp41 for cleavage-independent trimer assembly (8). Although NFL2P improves yield and stability, its broad applicability remains constrained. The UFO design is a native-like engineering method for HIV-1 Env trimers. It eliminates the dependency on enzymatic cleavage and incorporates optimizations targeting the membrane fusion process. Kong et al. (9) proposed this innovative design, which omits the I559P mutation and replaces the furin cleavage site with variable-length linkers. This innovation enhances the universal applicability and stability of the Env trimers across diverse HIV-1 strains. However, the purification of correctly folded trimers is quite complex.

Parallel innovations have driven respiratory syncytial virus (RSV) vaccine development, where NAbs primarily target metastable prefusion F (pre-F) trimers (45). Seminal work introduced the DS-Cav1 immunogen, combining disulfide mutations (S155C and S290C) and cavity-filling substitutions (S190F and V207L) to stabilize pre-F (10). Disulfide bond engineering suppresses dynamic fluctuations in key regions during conformational transitions, while bulky side-chain substitutions fill cavities caused by suboptimal hydrophobic packing in the prefusion state. This dual strategy restricts transitional spatial freedom and enhances thermodynamic stability. This design results in NAb titers exceeding protective thresholds in pre-clinical models. A recent study identified an optimized construct, which incorporated T103C–I148C disulfide bonds, S190I cavity-filling, and D486S charge-neutralizing mutations and demonstrated enhanced efficacy in phase III clinical trials (46). Notably, the nanoparticle display of DS-Cav1 on I53-50 scaffolds enhanced immunogenicity by 10-fold, an effect attributed to its spatial organization (11). This structure-based antigen design, combined with nanoparticle delivery to achieve broad-spectrum immunity, is now a strategy extended to EBOV, SARS-CoV-2, and HIV vaccine development (47–49).

The COVID-19 pandemic spurred rapid advances in structural vaccinology. SARS-CoV-2‘s spike (S) protein undergoes structural rearrangement to mediate viral-cell membrane fusion, but post-fusion S alone fails to elicit robust immunity. Corbett et al. (16) engineered a prefusion-stabilized S protein by introducing two proline mutations (2P), which effectively “lock” its conformation to preserve neutralizing epitopes. At present, the S-2P strategy has been utilized in multiple vaccines, including the mRNA vaccines by Moderna and BioNTech, the adenovirus vector vaccine from Johnson & Johnson, and the recombinant protein vaccine by Novavax. This technology has elicited more durable and potent immune responses. Two doses of the BNT162b2 (BioNTech) and mRNA-1273 (Moderna) vaccines provided 94.8% and 94.1% protection against COVID-19, respectively (17, 18). Based on S-2P, Hsieh et al. (19) further introduced four proline mutations (S-6P). These additional proline mutations further stabilize the prefusion conformation of the S protein and improve its expression level and stability.

The conformational dynamics of viral surface proteins helps evade NAbs. Therefore, stabilizing antigens in prefusion conformation depends on three strategies: covalent bond stabilization (e.g., disulfide bonds), steric hindrance optimization (e.g., hydrophobic cavity filling, charge neutralization, and proline substitutions), and cleavage site modification (e.g., eliminating cleavage-dependent transitional states). Emerging computational tools, including AlphaFold3, Colab Design, and Rosetta, enable rational design of prefusion-stabilized antigens for other enveloped viruses, such as the prefusion conformation of EBOV GP protein and the F proteins from Hendra virus (HeV), Nipah virus (NiV), human metapneumovirus (HMPV), and human parainfluenza virus (HPIV) (12–15, 20).

### Epitope-focused design: directing immune recognition toward conserved determinants

While stabilizing antigens in specific conformations boosts immunity, viral variants necessitate vaccines that target conserved regions across strains. Epitope-focused design directs immune responses to these invariant sites, sharpening antibody precision and reducing ineffective reactions. Multiple immune-focusing strategies have been developed to steer immune recognition toward defined antigenic regions, including epitope masking (glycosylation to occlude immunodominant regions), epitope scaffolding (transplanting epitopes onto stable scaffolds for enhanced immunogenicity), and antigen resurfacing (reducing non-target epitope immunogenicity by site-directed mutagenesis) (21). Building on this concept, Hauser et al. (22) harnessed the power of glycosylation engineering and epitope scaffolding in synergy to achieve broad-spectrum neutralization against SARS-CoV-2 and related coronaviruses. Beyond masking immunodominant regions, directing responses toward cryptic but conserved epitopes presents unique challenges. These are addressed by strategies, such as the protect-modify-deprotect (PMD), a three-step process: (1) protection of the target epitopes using bNAbs, (2) covalent modification of exposed lysine residues with PEG-NHS, and (3) deprotection of the target epitope by dissociation of NAb (23). This innovative approach leverages PEGylation and chemical masking effects to redirect immune recognition toward the previously hidden epitopes. The complexity of covalent modification and the process of antibody removal increases manufacturing costs and introduce risks of residual reagent toxicity. Moderna’s recent clinical trials of an mRNA-based RSV vaccine in infants revealed more severe respiratory infection symptoms compared to the placebo group (50). This situation underscores RSV-specific risks, such as fusion protein dominance or age-related immune responses. To address these challenges, current strategies focus on epitope engineering, exemplified by the LC2DM approach, which involves truncating immunodominant regions to remove non-protective epitopes while retaining key neutralizing targets. This design enhanced Th1 polarization and achieved durable NAb titers (> 84 days), though requiring careful validation to ensure conserved epitopes remained effectively recognizable by immune cells (24). Zhao et al. (25) achieved broad-spectrum protection against variants by displaying 16 copies of a conserved epitope of SARS-CoV-2 on a tailored horseshoe-shaped RNH1 scaffold, though the induced antibodies showed limited neutralization, potentially due to the epitope’s cryptic nature and suboptimal spatial density.

Two major obstacles in epitope-based vaccine design are mimicking the natural epitope structure and enhancing epitope immunogenicity. Selecting conserved B-cell and T-cell epitopes for vaccine development simplifies antigen design and enhances overall protective efficacy. Computational design tools are widely employed in the prediction of epitope vaccines (51). Sharma et al. (26) developed a universal multi-epitope subunit vaccine using immunoinformatics approaches that synergistically activates innate, cellular, and humoral immune responses. Furthermore, ConFormer-based epitope predictors (CFEP) have been developed to predict binding affinity of epitopes to both HLA classes I and II molecules. Vaccines designed using this approach effectively elicit T-cell responses and, by targeting conserved viral regions, exhibit broad protection against diverse variants (52). However, epitopes are usually small in size and thus less immunogenic. One of the promising strategies is to display the epitopes on the surface of nanoparticles. Many nanoparticles have been proven to be able to accommodate foreign epitopes without destroying particle assembly (53). The nanoparticle vaccine, engineered by displaying conserved epitopes of pre-existing NAbs on ferritin (CePnF), demonstrated robust induction of humoral, cellular, and mucosal immune responses while conferring broad-spectrum protection against diverse SARS-CoV-2 variants (54). However, epitope density and spacing on nanoparticles must be optimized to prevent steric hindrance, a challenge that requires iterative structural modeling and experimental validation. Simultaneously, multi-epitope tandem fusion may disrupt the protein scaffold’s hydrophilicity, leading to insoluble inclusion bodies, while also requiring avoidance of immunodominance imbalance. Structural modeling can predict optimal antigen combinations to maximally expose key immune epitopes. Moreover, the capacity of bNAbs to offer escape-resistant and long-lasting antiviral protection highlights the crucial role of structural characterization of their cognate epitopes in optimizing the design of pan-variant vaccines. Recent studies identified numerous bNAbs against various pathogens (55–57). The structural and functional characterization of these bNAbs has laid a path to innovative vaccine strategies. While bNAbs typically target conformational epitopes, *de novo* protein design enables engineered immunogens that mimic bNAb mechanisms, eliciting broad-spectrum protection against rapidly evolving pathogens. Recently, David Baker’s team computationally engineered a protein named nTrimer1 that potently neutralizes diverse MERS strains and demonstrated exceptional prophylactic efficacy in mouse models (58). This *de novo* design exemplifies the remarkable potential of computational protein design.

Vaccine design targeting conserved epitopes must balance two objectives: maximizing immunogenicity and minimizing viral escape. The glycan shield of HIV-1 Env exemplifies this challenge: it serves as a critical escape mechanism, yet complex glycopeptide epitopes underpin bNAb breadth. To address this paradox, attention should be directed toward functionally invariant regions, such as the CD4-binding loop and fusion peptide, where mutations severely impair viral fitness. Designing immunogens that expose these sites can force the virus to choose between “escape” and “inactivation”. Sequential immunization targeting multiple epitope clusters can induce antibodies against diverse regions, thereby preventing escape due to single-epitope mutations (59, 60).

### Consensus sequence engineering: capturing pan-viral shared features

Epitope-focused design enhances antibody specificity through immune-guided technologies. However, its reliance on predefined epitopes has driven researchers to investigate a critical question: How can we systematically uncover cross-subtype conserved elements at the viral genomic level? Consensus sequence engineering provides a solution via multiple sequence alignment-based mining of evolutionarily conserved motifs. Computationally optimized broadly reactive antigens (COBRA) represent the dominant methodology. This strategy has been extended to develop broad-spectrum vaccines against multiple pathogens, including influenza virus, dengue virus, SARS-CoV-2, NiV, and Chikungunya virus (27–31). Consensus sequence-based antigen design holds great promise for preventing emerging infectious diseases. Using an evolutionary clustering algorithm, Zhao et al. (32) identified conserved SARS-CoV-2 mutation sites and evolutionary patterns. The resulting S_pan_ antigen targets the five most frequent mutations, all of which persist in subsequently emerged Omicron subvariants, demonstrating strong conservation and foresight.

Despite significant progress, consensus-based vaccine development faces challenges. For influenza viruses, the rapid mutation rate and antigenic drift complicate the prediction of evolutionarily stable consensus sequences, thereby gradually diminishing vaccine efficacy over time. Notably, accurate prediction of consensus sequences depends critically on the viral database: larger and more diverse databases yield greater precision. Moreover, the database must represent currently circulating viruses to yield meaningful computational results. Furthermore, host genetic diversity introduces another hurdle: varied genetic backgrounds influence vaccine-induced immune responses, resulting in variable efficacy across species. This complicates control efforts for viruses capable of cross-species transmission. Pre-existing immunity from prior exposures (acquired from prior infection or vaccination) may interfere with consensus vaccine immunogenicity, limiting immune activation in pre-immune populations. This highlights the intricacies of designing personalized or universal vaccines. Critically, rational vaccine design requires not only advanced sequence analysis but also deep insights into viral evolution and host immunity. To advance this field, future efforts must integrate computational biology, structural virology, and systems immunology to streamline vaccine development.

### Chimeric antigen design: integrating immunodominant domains from heterologous strains

Chimeric antigen design represents a pivotal strategy to counteract viral variations. To advance broad-spectrum vaccines, it integrates key antigenic epitopes from divergent viral strains into a unified immunogen through two principal technical paradigms: epitope transplantation and domain substitution. Transplanting neutralizing epitopes from highly variable viruses onto conserved backbones can overcome genotype restrictions. For instance, transferring neutralizing epitopes from the VP1 surface loops of human norovirus (HuNoV) GII.4 to the GI.1 backbone successfully induced cross-reactive antibodies against both genotypes, demonstrating the critical role of epitope-backbone compatibility in broad immune coverage (33). According to Guthmiller et al. (34), the chimeric hemagglutinin (HA) vaccination reshaped the long-lived HA-specific B-cell repertoire and induced a convergence of B cells against multiple conserved and protective epitopes of HA. Vaccine design requires balancing immunodominance to target multiple conserved epitopes for broader protection. Broecker et al. (35) engineered “mosaic” HAs by replacing immunodominant antigenic sites in H3 HA with conserved epitopes derived from avian influenza HAs, thereby achieving antigenic silencing of variable regions. This approach overcomes seasonal vaccine limitations by refocusing immunity toward conserved epitopes, demonstrating broad cross-reactivity across H3 strains and conferring heterologous protection against antigenically drifted viruses. Multivalent antigens constructed through domain fusion enhance variant coverage. A representative case is the heterodimer formed by fusing SARS-CoV-2 prototype strain and beta variant receptor-binding domains (RBDs), which elicited bNAbs against alpha, beta, delta, and omicron via spatial synergy effects. Further incorporating beta and omicron mutations into alpha coronavirus backbones has achieved cross-variant protection and provided a pre-adaptive framework to counter viral evolution (36–38). The modularity of chimeric antigen design enables diverse applications beyond single-pathogen scenarios. For example, hybrid antigens combining the influenza HA stalk domains and the SARS-CoV-2 RBDs, as reported by Li et al. (39), induced dual NAbs against both viruses, highlighting the potential of chimeric antigen design for broad-spectrum protection. Despite the significant advantages that chimeric antigens offer, this approach also faces several challenges that impact its efficacy. First, the physicochemical stability of multi-domain fusion proteins can be compromised due to structural incompatibilities between heterologous domains. Second, immune dominance hierarchies among epitopes may suppress responses to subdominant targets, and careful safety evaluation is required for the novel antigen created by fusion. These limitations underscore the need for computational modeling and structural validation to ensure epitope accessibility and native-like folding. Additionally, for vector-based vaccines, the capacity to accommodate multiple antigens must also be considered.

Chimeric antigens can incorporate immunomodulatory molecules, such as CXCL13, CCL3, XCL1, or GM-CSF (40–44). These molecules stimulate dendritic cells (DCs) and recruit T follicular helper (Tfh) cells and germinal center (GC) B cells. This process promotes GC formation and non-specifically enhances immune responses. Critically, GC responses require stringent regulation to prevent autoantibody production and the development of systemic autoimmune diseases, while the chimeric construct’s safety requires comprehensive evaluation (61). Furthermore, spatial conformation disruptions during chimeric antigen construction may weaken effectiveness. Despite these challenges, chimeric design represents a powerful platform for developing next-generation broad-spectrum vaccines. Advances in structural biology, immunoinformatics, and synthetic biology will refine this strategy to better address viral evolution and pandemic threats.

## NANOPARTICLE PLATFORMS FOR BROAD-SPECTRUM IMMUNITY: VALENT ENGINEERING AND IMMUNE ENHANCEMENT

**Fig 2:**
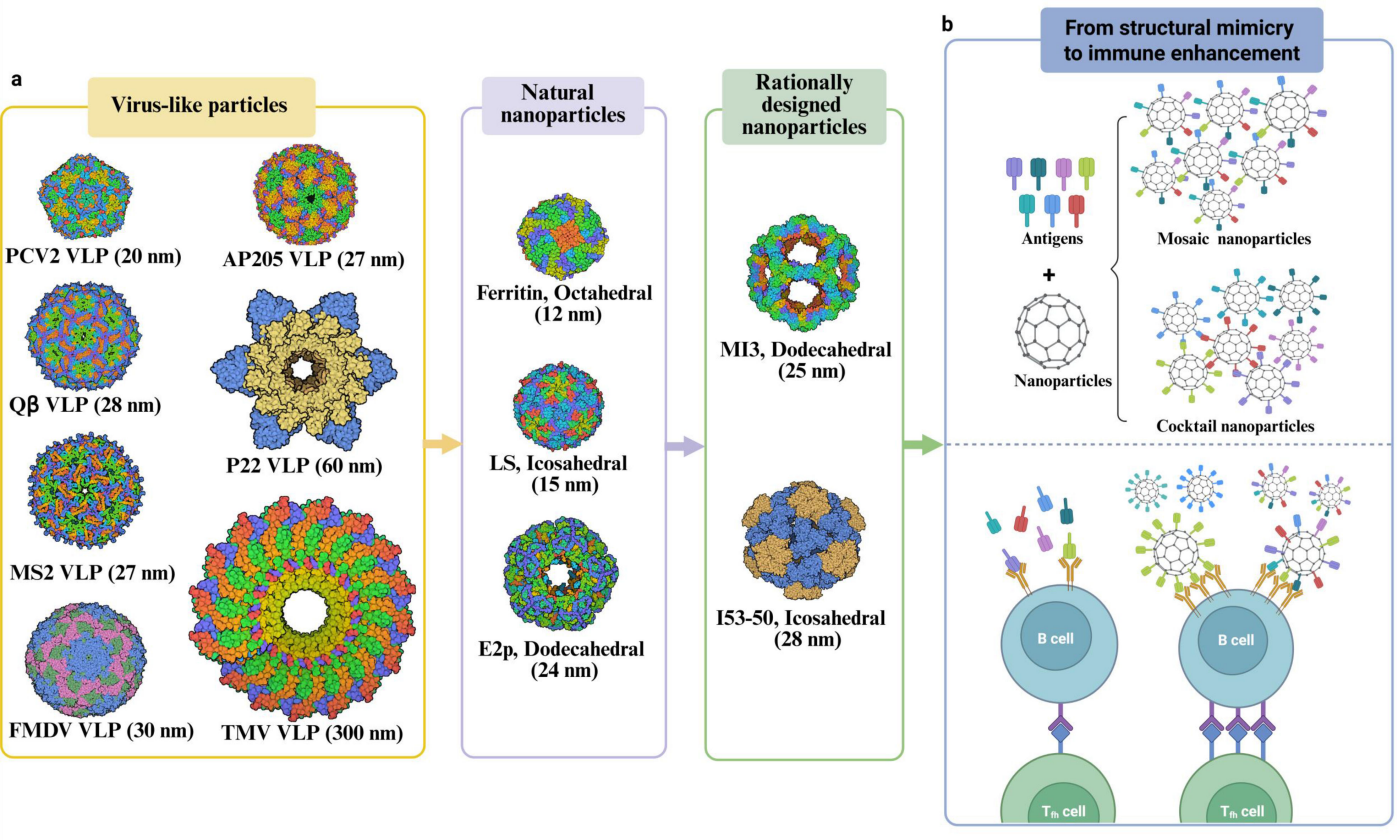
Protein-based nanoparticles: types and multivalent display strategies. (**a**) Representative categories of nanoparticles. Their structures were constructed by using the Protein Data Bank (PDB) ID codes, including protein-based platforms [e.g., VLPs derived from PCV2 (*6OLA*), FMDV (*7ENP*), MS2 (*6RRS*), P22 (*8EB7*), Q*β* (*7LGE*), TMV (*6RLP*), ferritin (*1MFR*), LS (*4*V7G), AP205 (*5LQP*), E2p (*1B5S*), MI3 (*7B3Y*), and I53-50 (*7SGE*)]. (**b**) Two nanoparticle delivery strategies to enhance immune responses: mosaic nanoparticles integrate diverse antigenic components within a single structural entity, whereas cocktail nanoparticles achieve synergistic effects through simultaneous delivery of multiple nanoparticle types each displaying a single antigen. Compared to single soluble antigens, multivalent antigen arrays on nanoparticles can cross-link multiple B-cell receptors to enhance antigen processing and presentation while simultaneously strengthening interactions with follicular helper T cells, thereby resulting in higher and longer-lasting antibody levels.

The successful implementation of these antigen designs critically depends on synergistic optimization with advanced delivery systems. For instance, the integration of structurally stabilized antigens with surface-displayed nanoparticles can synergistically enhance immune responses, while multi-epitope chimeric antigens require modular platforms to maximize immunogenicity. When the antigen spacing on nanoparticle surfaces approximates the optimal B-cell receptor (BCR) binding distance (~10 nm), multivalent antigens can simultaneously engage multiple BCRs, triggering potent B-cell activation signals. Concurrently, nanoparticles within this size range (20–200 nm) can drain to lymph nodes and be presented to resident DCs (62). In the following sections, we systematically categorize nanoparticle types (see Fig. 2a for representative structures), elucidate their distinctive characteristics, and evaluate their applications in enhancing immune protection. A detailed summary of the utilization of different nanoparticles for inducing broad-spectrum immune protection is provided in Table 2.

**TABLE 2:** Comparative analysis of nanoparticle vaccine platforms for broad-spectrum protection against viral infections

Vaccine platforms	Pathogens	Antigen design	Humoral immune response	Cellular immune response	Viral challenge protection	References
VLP	Seasonal influenza virus, HuNoV, sarbecoviruses, CSFV, and IAV	Displaying different antigens/epitopes on the VLP surface; transplanting neutralizing epitopes from variable viruses onto conserved backbones forms mosaic nanoparticles	Robust cross-reactive antibodies	Robust cross-reactive T-cell responses	Reduced viral loads	(33, 53, 63–70)
Ferritin	SARS-CoV-2 (Delta, WIV04, Omicron), and ASFV	Displaying conserved epitopes of various viral proteins on the surface of ferritin; targeting DCs	Antibodies with cross-reactivity and neutralizing activity	Potent and cross-reactive cellular immune responses	Complete protection	(41, 54, 71–73)
MI3	SARS-CoV-1/2 and HIV-1	Displaying multiple distinct antigens/epitopes on mosaic nanoparticles surface; glycosylation to occlude immunodominant regions; stabilizing the viral protein in the prefusion conformation	Enhanced antibody responses to mismatched strains	Not assessed	Not assessed	(48, 74–76)
I53-50	RSV, EBOV, SUDV, SARS-CoV-2, and HIV-1	Stabilizing the viral protein in the prefusion conformation; constructing mosaic/cocktail nanoparticle vaccines	Potent neutralizing and cross-reactive antibodies	Significant activation of CD4^+^ and CD8^+^ T cells	Protection against lethal challenge	(11, 47, 49, 77)
LNP	SARS-CoV-2 and SARS-like zoonotic coronavirus	Constructing chimeric antigen; co-delivering with STING agonist	Cross-reactive IgG antibodies against multiple variants	Robust Th1/Tc1 responses (IFN-γ and IL-2) and CD8^+^ T-cell activation	Complete protection inmice/hamsters	(37, 38)
mRNA-launched VLP	ZIKV, RABV, SARS-CoV-2, and HIV-1	Self-assembly of mRNA-encoded proteins into VLPs; fusing mRNA-encoded target proteins with nanoparticle-forming components; co-delivering mRNA-encoded target proteins and structural helper proteins or EABR	NAbs against various variants; durable antibody-mediated protection	Multifunctional CD8^+^ and CD4^+^ T-cell responses	Complete protection	(78–83)
VLP-encapsulated mRNA	SARS-CoV-2 and HSV-1	Integrating MS2 stem-loop structures into mRNA; targeting DCs	Cross-neutralizing IgG antibodies; durable responses up to 9 months	Enhanced T-cell-mediated immunity, generating more IFN-γ, TNF-α, and IL-2	Reduced the viral load	(84)

### Structural mimicry: virus-like particles (VLPs), natural and rationally designed protein nanoparticles

VLPs represent an attractive vaccine development platform due to their structural mimicry of native viruses, combined with the elimination of risks inherent in live vaccines. The rigid, repetitive surface structure of VLPs, a potent geometric pathogen-associated structural pattern (PASP), facilitates BCR cross-linking while enabling efficient natural IgM binding and C1q complement fixation, thereby promoting their deposition on follicular dendritic cells (FDCs) (85, 86). This activates potent immunity, as exemplified by HPV VLPs with L1 pentamers, which achieve a cervical cancer prevention rate of > 90% (63). VLPs derive from diverse sources, such as animal viruses, bacteriophages, and plant viruses (87, 88). In veterinary medicine, VLPs have shown promise in protection against a range of animal infectious diseases. For example, PCV2-based VLPs have been used to develop vaccines against PCV2 (64), while FMDV VLPs have induced long-lasting immune responses (65). Both VLP-based vaccines have been implemented in field disease control programs. Swine influenza A virus VLPs provide cross-protection against different strains (66). Notably, structurally diverse VLPs serve as versatile platforms for multivalent antigen presentation, exemplified by PCV2 VLPs displaying classical swine fever virus (CSFV) E2 protein or porcine reproductive and respiratory syndrome virus (PRRSV) neutralizing epitopes, and porcine parvovirus (PPV) VLPs presenting T- and B-cell epitopes of FMDV VP1 within surface loops—all eliciting robust dual-pathogen immunity (67, 68). These VLPs can display foreign antigens for bivalent vaccines. Bacteriophage-based VLPs (P22, Qβ, MS2, and AP205) and plant virus-derived VLPs (TMV and PapMV) exhibit stability and immunogenicity, advancing vaccine design (89–94). VLP production platforms include bacterial, yeast, insect cell, mammalian cell, plant cell, and cell-free systems. Bacterial systems offer cost-effective scalability (producing approximately 30% of current VLPs) but lack eukaryotic post-translational modifications (PTMs) and require stringent endotoxin control, partially mitigated by engineered endotoxin-free *E. coli*. Yeast systems allow limited glycosylation, but VLP extraction risks denaturation. Insect cells support eukaryotic PTMs yet face purification difficulties due to baculovirus contamination. Mammalian cells enable complex PTMs yet have high costs and low yields. Plant systems are rapid and low cost but lack mammalian-like glycosylation and have low productivity. Cell-free systems achieve high yields quickly but are limited by poor scalability and high costs (95, 96).

Ferritin serves as a structurally stable and extensively validated biomimetic platform, offering a viable alternative to VLPs despite its smaller size and simpler subunit architecture. As the most studied non-viral protein nanoparticle, ferritin comprises eight subunits with three-fold symmetry and functions as a structural scaffold for trimeric antigen presentation. Key pathogenic viral antigens (e.g., influenza HA, SARS-CoV-2 S, and RSV F) adopt functional trimeric structures. The strategic fusion of these antigenic molecules to ferritin enables the assembly of trimers that closely mimic their native structural architectures. Currently, two ferritin-based influenza vaccines have undergone Phase I clinical evaluation (NCT03186781 and NCT03814720), both of which have demonstrated the ability to induce the production of bNAbs (71, 72). Lumazine synthase (LS) is an enzyme involved in riboflavin biosynthesis, characterized by a highly symmetric icosahedral structure formed by the self-assembly of 60 subunits (97). LS exhibits high thermal stability (tolerating elevated temperatures) and resistance to degradation in physiological environments, making it suitable as a long-acting antigen carrier. When the VP8 protein of rotavirus was fused with LS nanoparticles, it was found that the monomeric VP8 on the nanoparticles exhibited denser and more closely spaced arrangements compared to the dimeric VP8 on native viral particles (98). However, LS nanoparticles fused with full-length HIV gp120 or gp140 failed to assemble. In contrast, dihydrolipoyl transacetylase protein (E2p), which forms a hollow dodecahedral 60-mer structure through the self-assembly of 20 trimers, successfully displayed HIV gp120 or gp140 and enhanced the breadth and durability of NAbs (99).

Natural protein assemblies inspire the engineering of programmable nanoparticles. Computationally designed architectures recapitulate native structural symmetry, self-assembly properties, and functional compartmentalization, thereby enabling precise control of antigen density, inter-epitope spacing, and spatial organization to optimize immune recognition. For example, I53-50 nanoparticles are a two-component protein complex, which is assembled *in vitro* by the icosahedral trimers I53-50A and the dodecahedral pentamers I53-50B (100). The I53-50 nanoparticle demonstrated enhanced immunogenicity when displaying RSV prefusion F proteins. By computationally simulating antigen spacing at 15 nm, which matches the optimal distance for BCR crosslinking, this configuration resulted in a 10-fold increase in NAbs compared to monomeric protein formulations (11). The I53-50-based SARS-CoV-2 recombinant protein nanoparticle vaccine GBP510 has entered clinical trials (NCT04750343) (77). Moreover, Bruun et al. (74) developed MI3, a mutant variant of the I301 residue on the 2-keto-3-deoxy-phosphogluconate (KDPG) aldolase from the hyperthermophilic bacterium *Thermotoga maritima*, which self-assembles into a porous dodecahedral structure composed of 60 subunits. MI3 nanoparticles displayed eight distinct SARS-CoV-2 RBDs via SpyTag-SpyCatcher, eliciting protection against multiple sarbecovirus strains (75). The SpyTag-SpyCatcher system enables modular antigen exchange, allowing a single nanoparticle platform to accommodate diverse pathogens and dynamically update antigens in response to emerging genetic variants. However, in comparison to other platforms, MI3 is relatively new and has received less attention in terms of safety and efficacy; thus, further validation is needed. In brief, these engineered platforms match the immunogenicity of natural counterparts, with oligomeric structures tailored for multimeric antigens, enhancing vaccine potential.

### Broad-spectrum immunity via mosaic and cocktail nanoparticle vaccines

Mosaic nanoparticles and cocktail nanoparticles represent a promising strategy for broad-spectrum vaccine development, demonstrating superior NAb induction compared to monovalent counterparts. These strategies leverage distinct approaches: mosaic nanoparticles rely on spatial engineering to precisely arrange multiple antigens on a single particle, while cocktail nanoparticles achieve modular delivery by mixing different single-antigen particles (differences between these delivery strategies are illustrated in Fig. 2b). To address emerging SARS-CoV-2 variants and sarbecovirus spillovers, Cohen et al. designed mosaic-8 RBD nanoparticles displaying eight different sarbecovirus RBDs. The probability of two adjacent RBDs being the same is low for mosaic-8 RBD nanoparticles, an arrangement chosen to favor interactions with B cells whose receptors can cross-link between adjacent RBDs to use avidity effects to preferentially recognize conserved, but sterically occluded, classes 3, 4, and 1/4 RBD epitopes. Mosaic-8 nanoparticles enhance heterologous binding, protecting against sarbecovirus challenges in animal models and eliciting more broadly cross-reactive antibodies against conserved epitopes than homotypic RBD-only nanoparticles (75, 76, 101). Nevertheless, this study had its limitations, as it failed to assess cellular immunity and did not conduct formal transmission studies. Notably, studies have shown that intranasal delivery of the mosaic nanoparticle vaccine elicits a robust mucosal immune response, confers broad cross-protection against multiple SARS-CoV-2 sublineages (102), and provides durable immune protection (73). To unravel the immunological underpinnings behind the enhanced breadth of antibody response and superior protection against heterologous infection offered by mosaic nanoparticle vaccines, Liu et al. (103) delved into the matter. Analysis of the nanoparticle-induced BCR repertoire revealed the preferential expansion of the IGHV14-3:IGKV14-111 germline pairing by mosaic nanoparticles. These mAbs conferred broad cross-protection through recognition of a conserved cryptic epitope on RBDs across clades 1a, 1b, and 3 sarbecoviruses. Similarly, Liu et al. (69) designed mosaic nanoparticles using genetic algorithms to maximize coverage of potential T-cell epitopes present in circulating strains. These nanoparticles exhibited cross-reactivity against 15 of 16 tested strains and were effective against a swine influenza virus, highlighting the broader applicability of this approach beyond coronaviruses. However, technical limitations persist, particularly because the display of different antigens onto the nanoparticles is random, and their positions and proportions cannot be predetermined. The construction of a quartet nanoparticle by arranging SARS-CoV RBDs on a single polypeptide chain and displaying it on MI3 nanoparticle surfaces addresses the challenge of uncontrollable antigen proportion. Compared to the mosaic-8 vaccine, the quartet nanocages, despite containing fewer components, induced broad-spectrum antibodies that effectively neutralized both target viruses and variant strains (104).

In contrast, cocktail nanoparticle vaccines are created by mixing individual nanoparticles, each displaying identical antigens. This modular approach avoids the stochastic antigen display patterns inherent in mosaic designs while retaining the flexibility to combine different antigens. For instance, a cocktail vaccine co-displaying S protein antigens from phylogenetically distinct clade 1 sarbecoviruses has been shown to induce bNAb responses against not only SARS-CoV-2 variants but also SARS-CoV-1 and zoonotic bat sarbecoviruses capable of binding to the human ACE2 (70). While cocktail nanoparticles offer advantages in fabrication simplicity and characterization over mosaic nanoparticles, both platforms entail sophisticated manufacturing processes and materials, resulting in relatively high production costs.

### Synergistic mRNA-VLP integration: programmable multivalent antigen display for enhanced immune protection

Pfizer and Moderna leveraged mRNA platforms to unprecedentedly develop SARS CoV-2 vaccines within merely 11 months, demonstrating the exceptional rapid response capability and flexibility of mRNA in addressing pandemic emergencies. The platform’s core advantage lies in real-time *in vivo* protein expression, enabling dynamic antigen adaptation: by modifying mRNA sequences to match viral antigenic drift, researchers rapidly iterated vaccines to target emerging variants with precision. However, challenges, such as large molecular size, negative charge, and susceptibility to enzymatic degradation, hinder their cellular internalization and cytoplasmic delivery, necessitating advanced delivery systems. LNPs remain the gold standard for mRNA encapsulation, offering protection and enhanced cellular uptake (105). Nevertheless, a frequently overlooked challenge in LNP technology lies in the remarkably limited cytoplasmic release of nucleic acid payloads. LNPs are internalized by cells through endocytic pathways, followed by trafficking to early endosomes that subsequently mature into late endosomes and ultimately lysosomes (106). To achieve efficient delivery, nucleic acid carriers must escape into the cytoplasm prior to endosomal maturation into degradative lysosomal compartments. This critical yet inefficient process is termed endosomal escape. Current escape mechanisms are primarily attributed to two hypotheses: (1) ionizable lipid protonation in acidic endosomes induces membrane non-bilayer phase transitions, and (2) proton sponge effects trigger endosomal osmotic rupture (107). A deeper understanding of these mechanisms is essential for the rational design of next-generation delivery systems.

**Fig 3:**
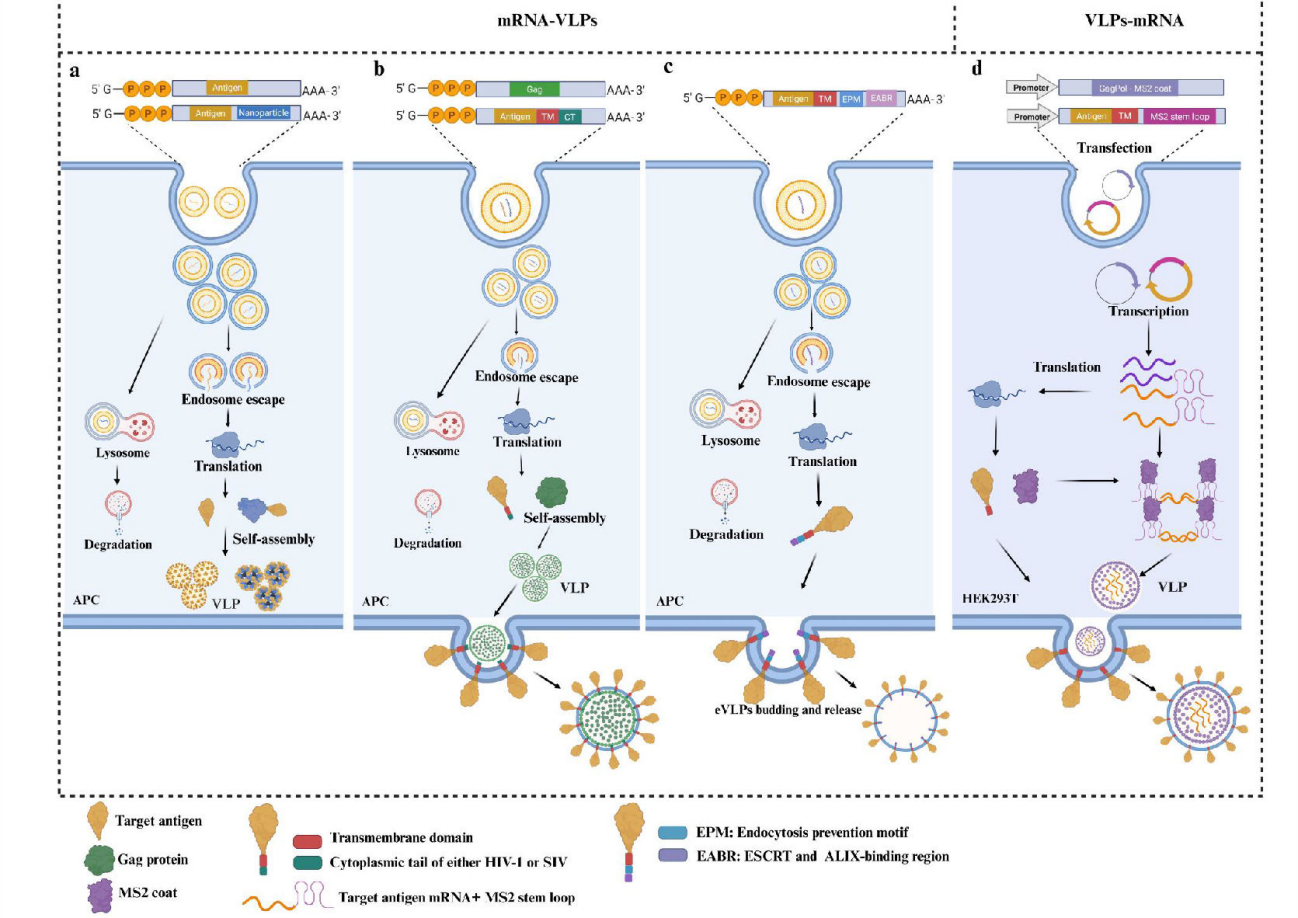
The synergistic strategy of mRNAs and VLPs. The mRNA-launched VLPs encompass the following approaches: (**a**) mRNA encodes self-assembling antigens forming VLPs *in vivo* or fusing with nanoparticles for surface display. (**b**) The target antigen (containing a transmembrane domain [TM] for membrane anchoring and a cytoplasmic tail [CT] sharing homology with the Gag protein of HIV or SIV) is co-delivered with Gag protein. During Gag VLP budding, the antigen binds to Gag via its CT, anchoring it onto the surface of enveloped VLPs (eVLPs). (**c**) Insertion of an endocytosis prevention motif (EPM) and an ESCRT/ALIX-binding region (EABR) into the CT of the target antigen. The EABR recruits ESCRT to drive the budding of the eVLPs from the cell membrane. (**d**) VLP-encapsulated mRNA via specific interactions between the MS2 stem-loop structure in the mRNA and the MS2 coat protein, enabling co-encapsulation of target antigen-encoding mRNA during MS2 VLP assembly.

In contrast, VLPs provide a self-assembling scaffold capable of multivalent antigen display, prolonged lymph node retention, and synergistic packaging of nucleic acids or adjuvants through their positively charged lumen (86). Recent advances have explored hybrid mRNA-VLP platforms to overcome limitations of conventional vaccines (illustrated in Fig. 3a through d). For example, LNP-encapsulated mRNA encoding Zika virus prM-E proteins self-assembled into VLPs *in vivo*, eliciting potent NAbs while minimizing cross-reactive antibodies to avoid antibody-dependent enhancement (ADE) of dengue virus infection (78). Similarly, the mRNA, encoding glycoprotein and matrix proteins of rabies virus to assemble VLPs *in vivo*, induces broader and longer-lasting NAbs than conventional inactivated vaccines (79). While these studies primarily focused on humoral immunity, Hendricks et al. (80) demonstrated that the mRNA-launched VLPs could induce robust CD8^+^ T cell responses alongside NAbs, offering protection against both matched and mismatched viral strains.

Recent engineering advances have expanded strategies for *in vivo* VLP formation. For viral proteins lacking self-assembly capability, fusion with self-assembling nanoparticles or co-expression with other viral proteins enables VLP generation. For instance, Zhang et al. co-delivered mRNAs encoding HIV-1 Env and SIV Gag, producing Env-displaying enveloped VLPs *in vivo* that induced bNAbs in non-human primates. This approach was further applied to SARS-CoV-2 mRNA-launched VLP design (81, 82). Hoffmann et al. (83) pioneered an endosomal sorting complex required for transport (ESCRT) pathway-driven VLP assembly strategy, eliminating dependency on other viral proteins. The key modification is fusing the cytoplasmic tail of the SARS-CoV-2 spike protein with an ESCRT- and ALG-2-interacting domain (EABR). This hijacks the ESCRT machinery, a cellular system that regulates membrane remodeling, to drive spontaneous VLP assembly and release from host cells (108). The mRNA-launched VLPs thus synergistically combine the multivalent antigen display capability of nanoparticles with the rapid adaptability of mRNA, eliciting stronger antibody responses than traditional Gag-dependent systems. This methodology establishes a versatile framework for membrane-bound antigen vaccines, expanding the scope of modular vaccine design. Collectively, these studies demonstrate enhanced synergy between mRNA and VLPs in eliciting durable humoral and cellular immunity.

VLPs serve not only as multivalent scaffolds for displaying foreign antigens but also package negatively charged nucleic acids or adjuvants via their positively charged inner surfaces, enhancing immune potency. Ling *et al*. (109) engineered lentiviral vectors based on the bacteriophage MS2 system, which leverages the specific interaction between the MS2 coat protein and stem-loop structures on mRNA to achieve co-packaging and delivery of Cas9 mRNA and a VEGFA-targeting gRNA. This innovative strategy effectively mitigates the off-target effects and immunogenicity concerns associated with prolonged Cas9 expression mediated by conventional viral vectors. Targeted delivery strategies further improve efficacy. Yin et al. (84) engineered mRNA-loaded VLPs decorated with the Sindbis virus glycoproteins, enabling DC-SIGN receptor-mediated dendritic cell targeting. Compared to non-targeted VLPs or LNPs, DC-targeted VLPs-encapsulated mRNA elicited superior and sustained adaptive immunity, highlighting the critical role of antigen-presenting cells in vaccine efficacy. Additionally, by enabling surface insertion of exogenous peptides and leveraging the properties of encapsulated mRNA, MS2 coat proteins facilitate the design of mRNA-peptide combination vaccines. This integrated approach presents a promising platform for combating complex viruses. Despite these advances, VLPs face two critical barriers. One is that VLPs often become trapped in endosomes and ultimately degraded after cellular uptake. Moreover, the confined interior space of VLPs poses limitations, leading to suboptimal or inefficient mRNA translation, thereby hampering the overall effectiveness. Future research should focus on the rational design and precise functionalization of VLPs to achieve dynamic regulation of their cellular entry and assembly states, thereby optimizing delivery efficiency.

### Surface-functionalized nanoparticles enable immune enhancement through precision targeting

**Fig 4:**
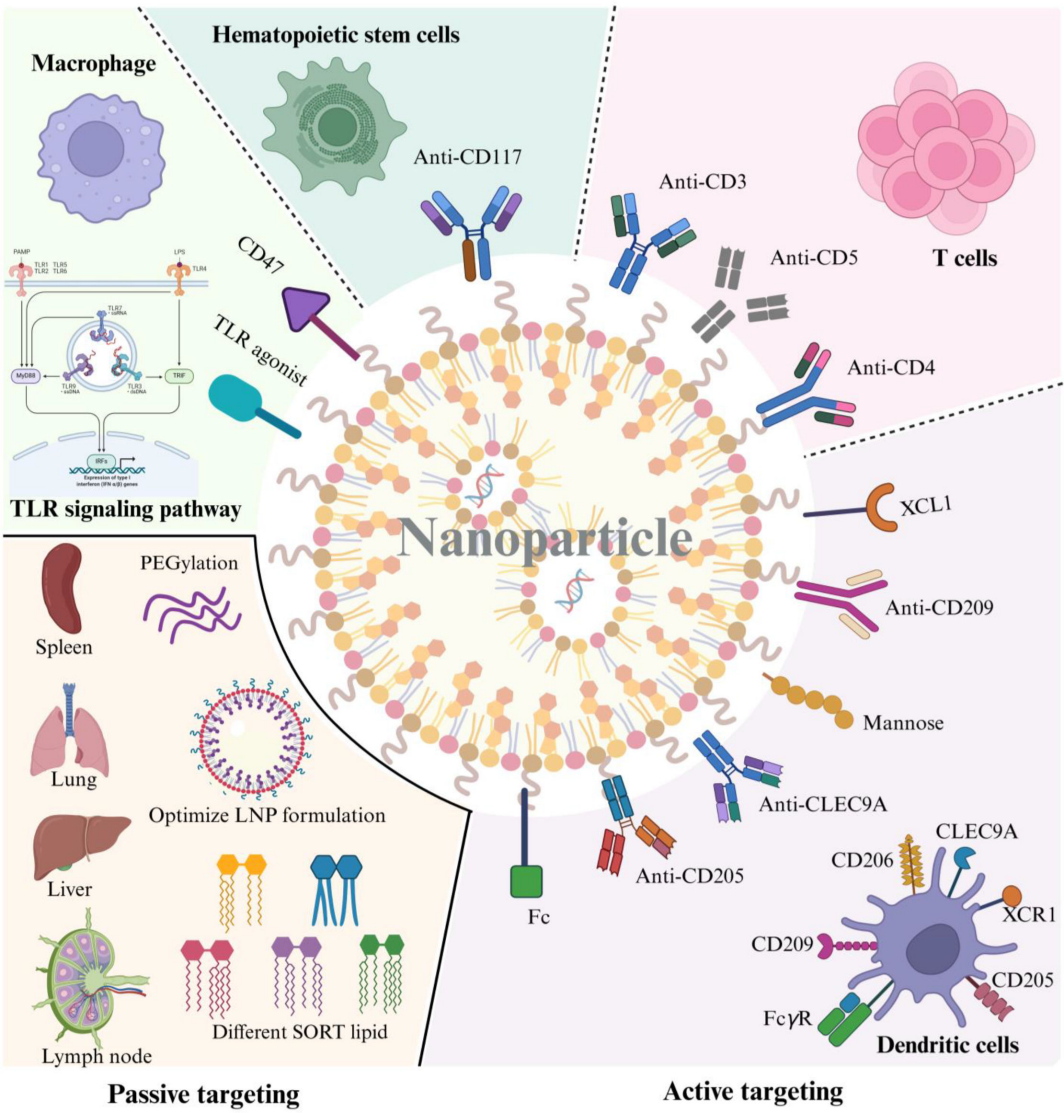
Surface functionalization of nanoparticles for targeted delivery. In active targeting, nanoparticles are modified with antibodies (e.g., anti-CD3/CD4/CD5 for T cells; anti-CD117 antibodies for hematopoietic stem cells), ligands, or antibodies (e.g., mannose, XCL1, anti-CD205, anti-CLEC9A, or anti-CD209 antibodies) to target specific cell surface receptors on DCs. Additionally, modifications by CD47 or a TLR agonist regulate cellular uptake or immune responses. For passive targeting, optimization of LNP formulation (e.g., insert different SORT lipids or introduce PEGylation) adjusts surface properties (charge, size) to enhance targeting efficiency toward organs, such as the spleen, lung, liver, and lymph nodes.

Nanoparticle surface engineering plays a pivotal role in optimizing nucleic acid delivery efficacy by tailoring physicochemical properties to enhance biocompatibility, stability, and tissue-specific targeting ability. This methodology enables organ-selective delivery, minimizes off-target distribution, and improves therapeutic precision (110, 111). As illustrated in Fig. 4, surface functionalization strategies are divided into active and passive targeting. Active targeting employs ligand-receptor interactions or antibody modification to promote precise targeting. For instance, anti-CD3/CD4/CD5 antibody–conjugated LNPs target T lymphocytes, while the CD117-conjugated LNPs efficiently target hematopoietic stem cells (HSCs) (112, 113). Complementary approaches include CD47-mediated evasion of phagocytic clearance and toll-like receptors (TLRs) agonist-functionalized nanoparticles that enhance immunostimulatory responses (114, 115). In contrast, passive targeting involves the optimization of LNP surface properties, including charge and size, through the adjustment of lipid composition to improve targeting efficacy (116). Notably, PEG critically influences extrahepatic targeting efficacy, and PEGylation of LNP will improve the LNPs’ stability and facilitate readily tissue penetration and targeting (117). Given the pivotal roles of DCs in adaptive immunity, particularly their ability to cross-present exogenous antigens via MHC-I, activating cytotoxic T cells (CTLs) and triggering antitumor or antiviral responses, this review specifically focuses on DC-targeted vaccine strategies.

Current DC-targeting strategies exploit multiple surface receptors, including Fc*γ* receptors (Fc*γ*Rs), lymphocyte antigen 75 (CD205), mannose receptor (CD206), DC-SIGN (CD209), C-type lectin domain family 9 member A (CLEC9A), and XC-chemokine receptor 1 (XCR1) (118). However, expression patterns of surface receptors often vary between species (119). Research has demonstrated that mannose-modified nanoparticles enhance CD8^+^ T-cell priming through improved DC uptake and antigen cross-presentation, while combinatorial approaches integrating STING agonists or PD-1 blockade synergistically amplify antitumor immunity (120). The glucose transporter 1 (Glut-1) is also a reliable target for delivering antigens to DCs, offering effective immunotherapy for various types of tumors (121). In the development of mRNA vaccines for influenza, SARS-CoV-2, and rabies, targeted modifications preserved post-lyophilization physical properties without compromising targeting specificity or immunogenicity (122, 123). Distinct immune polarization outcomes arise from targeting different DC surface receptors: while engagement of MHC-II molecules predominantly drives Th2-biased responses with IgG1 dominance, chemokine receptors CCR1/3/5 instead elicit mixed Th1/Th2 polarization accompanied by comparable IgG1 and IgG2a elevation. Conversely, XCR1 targeting induces robust Th1 polarization and cell-mediated immunity (124). These findings demonstrate that precision receptor targeting enables programmable control over Th1/Th2 bias, antibody isotype profiles, and effector cell differentiation, highlighting how strategic receptor selection governs the “quality” of adaptive immunity beyond merely enhancing response magnitude. While DC-targeted vaccines represent a paradigm shift from *ex vivo* approaches like sipuleucel-T, the first FDA-approved therapeutic cancer vaccine, which represents a landmark achievement in autologous DC-based immunotherapy (125), simplifies the process and potentially reduces costs.

However, there are some drawbacks, such as certain receptors not being exclusive to DCs; for instance, macrophages also express mannose receptors. Moreover, it has been reported that soluble mannose receptor levels are elevated in patients with a variety of inflammatory diseases (126). Thus, potential off-target effects need to be carefully evaluated when exploiting targets expressed on multiple cell types. Upon entering the bloodstream, nanoparticles are immediately opsonized by complement protein C3. This leads to their clearance by phagocytes, compromising targeting efficacy and triggering the release of anaphylatoxins (127). Pre-existing anti-PEG antibodies, despite PEG’s role in reducing RES clearance (128), can activate complement, compromising the integrity of PEGylated LNPs and causing cargo leakage or exposure (129). Conjugation chemistries pose challenges in antibody orientation control and characterization complexity, complicating manufacturing processes. Furthermore, studies demonstrate that these chemistries directly participate in activating the complement cascade of plasma proteins. Their activation of complement causes dramatic alterations in nanoparticle biodistribution (130). It should also be noted that surface-engineered NPs may not always function as intended *in vivo*. This is because various serum proteins rapidly and non-specifically adsorb onto the NP surface, forming a “protein corona”. This corona significantly masks conjugated targeting ligands and facilitates rapid clearance from systemic circulation by the mononuclear phagocyte system (131). Additionally, strategic selection of covalent vs. non-covalent modification methods must consider nanoparticle core properties, functional group reactivity, and end-product stability requirements. Most importantly, surface engineering critically dictates nanoparticle characteristics (hydrodynamic diameter, zeta potential, dissolution kinetics, and thermodynamic stability), which collectively govern biological outcomes, including cytotoxicity, immune responses, biodistribution, pharmacokinetics, and organ-specific accumulation.

## CONCLUSIONS AND PERSPECTIVES

The development of broad-spectrum vaccines represents not merely a technological advancement but a pivotal paradigm shift essential for combating rapidly evolving viral pathogens—moving decisively from a “reactive response to current strains” toward “proactive prevention of future threats”. Successful realization of this shift hinges primarily on breakthroughs in antigen design science, not merely on advancements in production technology. Innovations in antigen design are rooted in advances in scientific concepts and methodologies. While advances in production technology can be rapidly implemented, fundamental scientific discoveries require rigorous validation over time. Nanoparticles serve as versatile platforms for antigen delivery and immune enhancement. Their nanoscale size facilitates efficient uptake by APCs, while surface antigen multimerization enhances receptor-binding affinity and immune activation. This review highlights the transformative synergy between rational antigen design and nanoparticle delivery platforms, which collectively address the limitations of conventional strain-specific vaccines. Key advancements in structural vaccinology, epitope-focused engineering, consensus sequence optimization, and chimeric antigen design are the engines powering this transition, enabling the precise targeting of conserved viral regions and enhancing both the magnitude and breadth of immune responses. Concurrently, nanoparticle platforms, including spanning VLPs, natural nanoparticles, and computationally designed nanoparticles, enhance immunogenicity through multivalent antigen display, targeted delivery, and controlled release kinetics. Innovations such as mosaic or cocktail nanoparticles, mRNA-launched VLPs or VLPs-encapsulated mRNA, and surface-functionalized delivery further exemplify the versatility of these strategies in eliciting cross-reactive humoral and cellular immunity, laying the groundwork for proactive defense.

This paradigm shift necessitates tailoring vaccine design strategies to both the functional mechanisms of protective antibodies and the mechanisms by which specific antigens induce immunity for each disease. Future research focused on proactive prevention should prioritize: (1) Integrating artificial intelligence (AI) to develop precise antigen design. Machine learning techniques (e.g., deep learning and random forests) can predict conserved epitopes with high accuracy, quantify immunogenicity, simulate immune interactions, and optimize antigen stability by integrating multidimensional data on pathogen genomics, protein structures, and immune responses. This reduces reliance on empirical screening, accelerates the transition from laboratory to clinical applications, and significantly lowers development costs. These outcomes are enabled by an AI-powered predictive model that integrates viral evolutionary dynamics with host immune profiles, supported by a collaborative, multi-algorithm optimization framework synthesizing genomic data, immune response characteristics, and clinical information. This comprehensive system should encompass the entire vaccine development pipeline, from antigen screening and design to adjuvant selection and formulation refinement (51). For pathogens with undefined protective antigens, like African Swine Fever Virus (ASFV), existing vaccines are primarily live-attenuated types. While these elicit strong cellular immunity, they pose risks of reversion to virulence and biosafety concerns (132). Integrating machine learning holds significant potential here: models trained on ASFV structural biology, genomic, and immunology data can predict an antigen’s potential to activate both humoral and cellular immune responses. This identifies protective antigens—those that trigger cellular immunity and whose antibodies effectively inhibit viral replication. Designing diverse vaccines targeting these antigens for sequential immunization can thereby reshape the immune system for effective protection. However, data heterogeneity and limited availability constrain AI model performance, while substantial hardware requirements and restricted access to high-performance computing facilities further impede their application. (2) Innovative nanomaterials and delivery strategies can enhance immune activation. For instance, stimuli-responsive nanoparticles that release antigens upon encountering intracellular signals (e.g., pH changes and enzymatic activity) enable spatiotemporal control of antigen delivery (133). Emerging delivery systems, such as extracellular vesicles and hydrogels, show great potential for vaccine development (134, 135). Co-delivery of antigens with adjuvants (e.g., TLR/STING agonists) via nanoparticles further amplifies immune responses through synergistic activation (136). Optimizing modular platforms (e.g., SpyTag-SpyCatcher-functionalized MI3 nanoparticles) for rapid antigen swapping enables real-time updates against emerging variants. For broad application in vaccine development, the manufacturing processes of these nanomaterials require further refinement to enable cost-effective and large-scale production while maintaining their safety profiles. (3) Interdisciplinary integration is essential to bridge translational gaps. Combining computational biology, structural virology, and systems immunology would address the interplay between viral evolution, immune recognition, and delivery efficiency. By embracing and advancing these fronts, the vision of proactive, broad-spectrum protection against known pathogens and unforeseen future pandemic threats can be transformed from a scientific imperative into a practical reality.

## References

[1] Dye C. 2014. After 2015: infectious diseases in a new era of health and development. Philos Trans R Soc Lond B Biol Sci 369:20130426. doi:10.1098/rstb.2013.0426 24821913 PMC4024220

[2] Greenwood B. 2014. The contribution of vaccination to global health: past, present and future. Philos Trans R Soc Lond B Biol Sci 369:20130433. doi:10.1098/rstb.2013.0433 24821919 PMC4024226

[3] Wang Y, Wang D, Zhang L, Sun W, Zhang Z, Chen W, Zhu A, Huang Y, Xiao F, Yao J, et al.. 2021. Intra-host variation and evolutionary dynamics of SARS-CoV-2 populations in COVID-19 patients. Genome Med 13:30. doi:10.1186/s13073-021-00847-5 33618765 PMC7898256

[4] Li D, Sun C, Zhuang P, Mei X. 2024. Revolutionizing SARS-CoV-2 omicron variant detection: towards faster and more reliable methods. Talanta 266:124937. doi:10.1016/j.talanta.2023.124937 37481886

[5] Ward AB, Wilson IA. 2020. Innovations in structure-based antigen design and immune monitoring for next generation vaccines. Curr Opin Immunol 65:50–56. doi:10.1016/j.coi.2020.03.013 32387642 PMC7174181

[6] Nguyen NT, Le XT, Lee WT, Lim YT, Oh KT, Lee ES, Choi HG, Youn YS. 2024. STING-activating dendritic cell-targeted nanovaccines that evoke potent antigen cross-presentation for cancer immunotherapy. Bioact Mater 42:345–365. doi:10.1016/j.bioactmat.2024.09.002 39290338 PMC11406000

[7] Sanders RW, Derking R, Cupo A, Julien J-P, Yasmeen A, de Val N, Kim HJ, Blattner C, de la Peña AT, Korzun J, Golabek M, de Los Reyes K, Ketas TJ, van Gils MJ, King CR, Wilson IA, Ward AB, Klasse PJ, Moore JP. 2013. A next-generation cleaved, soluble HIV-1 Env trimer, BG505 SOSIP.664 gp140, expresses multiple epitopes for broadly neutralizing but not non-neutralizing antibodies. PLoS Pathog 9:e1003618. doi:10.1371/journal.ppat.1003618 24068931 PMC3777863

[8] Sharma SK, de Val N, Bale S, Guenaga J, Tran K, Feng Y, Dubrovskaya V, Ward AB, Wyatt RT. 2015. Cleavage-independent HIV-1 Env trimers engineered as soluble native spike mimetics for vaccine design. Cell Rep 11:539–550. doi:10.1016/j.celrep.2015.03.047 25892233 PMC4637274

[9] Kong L, He L, de Val N, Vora N, Morris CD, Azadnia P, Sok D, Zhou B, Burton DR, Ward AB, Wilson IA, Zhu J. 2016. Uncleaved prefusion-optimized gp140 trimers derived from analysis of HIV-1 envelope metastability. Nat Commun 7:12040. doi:10.1038/ncomms12040 27349805 PMC4931249

[10] McLellan JS, Chen M, Joyce MG, Sastry M, Stewart-Jones GBE, Yang Y, Zhang B, Chen L, Srivatsan S, Zheng A, et al.. 2013. Structure-based design of a fusion glycoprotein vaccine for respiratory syncytial virus. Science 342:592–598. doi:10.1126/science.1243283 24179220 PMC4461862

[11] Marcandalli J, Fiala B, Ols S, Perotti M, de van der Schueren W, Snijder J, Hodge E, Benhaim M, Ravichandran R, Carter L, Sheffler W, Brunner L, Lawrenz M, Dubois P, Lanzavecchia A, Sallusto F, Lee KK, Veesler D, Correnti CE, Stewart LJ, Baker D, Loré K, Perez L, King NP. 2019. Induction of potent neutralizing antibody responses by a designed protein nanoparticle vaccine for respiratory syncytial virus. Cell 176:1420–1431. doi:10.1016/j.cell.2019.01.046 30849373 PMC6424820

[12] Wong JJW, Paterson RG, Lamb RA, Jardetzky TS. 2016. Structure and stabilization of the Hendra virus F glycoprotein in its prefusion form. Proc Natl Acad Sci USA 113:1056–1061. doi:10.1073/pnas.1523303113 26712026 PMC4743799

[13] Loomis RJ, Stewart-Jones GBE, Tsybovsky Y, Caringal RT, Morabito KM, McLellan JS, Chamberlain AL, Nugent ST, Hutchinson GB, Kueltzo LA, Mascola JR, Graham BS. 2020. Structure-based design of Nipah virus vaccines: a generalizable approach to paramyxovirus immunogen development. Front Immunol 11:842. doi:10.3389/fimmu.2020.00842 32595632 PMC7300195

[14] Hsieh CL, Rush SA, Palomo C, Chou CW, Pickens W, Más V, McLellan JS. 2022. Structure-based design of prefusion-stabilized human metapneumovirus fusion proteins. Nat Commun 13:1299. doi:10.1038/s41467-022-28931-3 35288548 PMC8921277

[15] Stewart-Jones GBE, Chuang G-Y, Xu K, Zhou T, Acharya P, Tsybovsky Y, Ou L, Zhang B, Fernandez-Rodriguez B, Gilardi V, et al.. 2018. Structure-based design of a quadrivalent fusion glycoprotein vaccine for human parainfluenza virus types 1–4. Proc Natl Acad Sci USA 115:12265–12270. doi:10.1073/pnas.1811980115 30420505 PMC6275507

[16] Corbett KS, Edwards DK, Leist SR, Abiona OM, Boyoglu-Barnum S, Gillespie RA, Himansu S, Schäfer A, Ziwawo CT, DiPiazza AT, et al.. 2020. SARS-CoV-2 mRNA vaccine design enabled by prototype pathogen preparedness. Nature 586:567–571. doi:10.1038/s41586-020-2622-0 32756549 PMC7581537

[17] Baden LR, El Sahly HM, Essink B, Kotloff K, Frey S, Novak R, Diemert D, Spector SA, Rouphael N, Creech CB, et al.. 2021. Efficacy and safety of the mRNA-1273 SARS-CoV-2 vaccine. N Engl J Med 384:403–416. doi:10.1056/NEJMoa2035389 33378609 PMC7787219

[18] Skowronski DM, De Serres G. 2021. Safety and efficacy of the BNT162b2 mRNA COVID-19 vaccine. N Engl J Med 384:1576–1577. doi:10.1056/NEJMc2036242 33596348

[19] Hsieh CL, Goldsmith JA, Schaub JM, DiVenere AM, Kuo HC, Javanmardi K, Le KC, Wrapp D, Lee AG, Liu Y, Chou CW, Byrne PO, Hjorth CK, Johnson NV, Ludes-Meyers J, Nguyen AW, Park J, Wang N, Amengor D, Lavinder JJ, Ippolito GC, Maynard JA, Finkelstein IJ, McLellan JS. 2020. Structure-based design of prefusion-stabilized SARS-CoV-2 spikes. Science 369:1501–1505. doi:10.1126/science.abd0826 32703906 PMC7402631

[20] Rutten L, Gilman MSA, Blokland S, Juraszek J, McLellan JS, Langedijk JPM. 2020. Structure-based design of prefusion-stabilized filovirus glycoprotein trimers. Cell Rep 30:4540–4550. doi:10.1016/j.celrep.2020.03.025 32234486 PMC7118701

[21] Musunuri S, Weidenbacher PAB, Kim PS. 2024. Bringing immunofocusing into focus. NPJ Vaccines 9:11. doi:10.1038/s41541-023-00792-x 38195562 PMC10776678

[22] Hauser BM, Sangesland M, St Denis KJ, Lam EC, Case JB, Windsor IW, Feldman J, Caradonna TM, Kannegieter T, Diamond MS, Balazs AB, Lingwood D, Schmidt AG. 2022. Rationally designed immunogens enable immune focusing following SARS-CoV-2 spike imprinting. Cell Rep 38:110561. doi:10.1016/j.celrep.2022.110561 35303475 PMC8898741

[23] Bruun TUJ, Do J, Weidenbacher P-B, Utz A, Kim PS. 2024. Engineering a SARS-CoV-2 vaccine targeting the receptor-binding domain cryptic-face via immunofocusing. ACS Cent Sci 10:1871–1884. doi:10.1021/acscentsci.4c00722 39463836 PMC11503491

[24] Lin M, Yin Y, Zhao X, Wang C, Zhu X, Zhan L, Chen L, Wang S, Lin X, Zhang J, Xia N, Zheng Z. 2025. A truncated pre-F protein mRNA vaccine elicits an enhanced immune response and protection against respiratory syncytial virus. Nat Commun 16:1386. doi:10.1038/s41467-025-56302-1 39910047 PMC11799228

[25] Zhao F, Zhang Y, Zhang Z, Chen Z, Wang X, Wang S, Li R, Li Y, Zhang Z, Zheng W, Wang Y, Zhang Z, Wu S, Yang Y, Zhang J, Zai X, Xu J, Chen W. 2025. Epitope-focused vaccine immunogens design using tailored horseshoe-shaped scaffold. J Nanobiotechnol 23:119. doi:10.1186/s12951-025-03200-9 PMC11834273 39966941

[26] Sharma S, Kumari V, Kumbhar BV, Mukherjee A, Pandey R, Kondabagil K. 2021. Immunoinformatics approach for a novel multi-epitope subunit vaccine design against various subtypes of Influenza A virus. Immunobiology 226:152053. doi:10.1016/j.imbio.2021.152053 33517154

[27] Allen JD, Ross TM. 2022. Bivalent H1 and H3 COBRA recombinant hemagglutinin vaccines elicit seroprotective antibodies against H1N1 and H3N2 influenza viruses from 2009 to 2019. J Virol 96:e0165221. doi:10.1128/jvi.01652-21 35289635 PMC9006891

[28] Sankaradoss A, Jagtap S, Nazir J, Moula SE, Modak A, Fialho J, Iyer M, Shastri JS, Dias M, Gadepalli R, et al.. 2022. Immune profile and responses of a novel dengue DNA vaccine encoding an EDIII-NS1 consensus design based on Indo-African sequences. Mol Ther 30:2058–2077. doi:10.1016/j.ymthe.2022.01.013 34999210 PMC8736276

[29] Yasmin S, Ansari MY, Pandey K, Dikhit MR. 2024. Identification of potential vaccine targets for elicitation of host immune cells against SARS-CoV-2 by reverse vaccinology approach. Int J Biol Macromol 265:130754. doi:10.1016/j.ijbiomac.2024.130754 38508555

[30] Lu M, Yao Y, Liu H, Zhang X, Li X, Liu Y, Peng Y, Chen T, Sun Y, Gao G, Chen M, Zhao J, Zhang X, Yin C, Guo W, Yang P, Hu X, Rao J, Li E, Wong G, Yuan Z, Chiu S, Shan C, Lan J. 2023. Vaccines based on the fusion protein consensus sequence protect Syrian hamsters from Nipah virus infection. JCI Insight 8:e175461. doi:10.1172/jci.insight.175461 37917215 PMC10795836

[31] Coirada FC, Fernandes ER, Mello L de, Schuch V, Soares Campos G, Braconi CT, Boscardin SB, Santoro Rosa D. 2023. Heterologous DNA prime-subunit protein boost with Chikungunya virus E2 induces neutralizing antibodies and cellular-mediated immunity. Int J Mol Sci 24:10517. doi:10.3390/ijms241310517 37445695 PMC10342165

[32] Zhao Y, Ni W, Liang S, Dong L, Xiang M, Cai Z, Niu D, Zhang Q, Wang D, Zheng Y, Zhang Z, Zhou D, Guo W, Pan Y, Wu X, Yang Y, Jing Z, Jiang Y, Chen Y, Yan H, Zhou Y, Xu K, Lan K. 2023. Vaccination with S_pan_, an antigen guided by SARS-CoV-2 S protein evolution, protects against challenge with viral variants in mice. Sci Transl Med 15:eabo3332. doi:10.1126/scitranslmed.abo3332 36599007

[33] Hou YN, Jin YQ, Zhang XF, Tang F, Hou JW, Liu ZM, Han ZB, Zhang H, Du LF, Shao S, Su JG, Liang Y, Zhang J, Li QM. 2023. Chimeric virus-like particles of human norovirus constructed by structure-guided epitope grafting elicit cross-reactive immunity against both GI.1 and GII.4 genotypes. J Virol 97:e0093823. doi:10.1128/jvi.00938-23 37792003 PMC10617407

[34] Guthmiller JJ, Yu-Ling Lan L, Li L, Fu Y, Nelson SA, Henry C, Stamper CT, Utset HA, Freyn AW, Han J, et al.. 2025. Long-lasting B cell convergence to distinct broadly reactive epitopes following vaccination with chimeric influenza virus hemagglutinins. Immunity 58:980–996. doi:10.1016/j.immuni.2025.02.025 40132593 PMC11981830

[35] Broecker F, Liu STH, Suntronwong N, Sun W, Bailey MJ, Nachbagauer R, Krammer F, Palese P. 2019. A mosaic hemagglutinin-based influenza virus vaccine candidate protects mice from challenge with divergent H3N2 strains. NPJ Vaccines 4:31. doi:10.1038/s41541-019-0126-4 31341648 PMC6642189

[36] Xu K, Gao P, Liu S, Lu S, Lei W, Zheng T, Liu X, Xie Y, Zhao Z, Guo S, et al.. 2022. Protective prototype-Beta and Delta-Omicron chimeric RBD-dimer vaccines against SARS-CoV-2. Cell 185:2265–2278. doi:10.1016/j.cell.2022.04.029 35568034 PMC9042943

[37] Martinez DR, Schäfer A, Leist SR, De la Cruz G, West A, Atochina-Vasserman EN, Lindesmith LC, Pardi N, Parks R, Barr M, Li D, Yount B, Saunders KO, Weissman D, Haynes BF, Montgomery SA, Baric RS. 2021. Chimeric spike mRNA vaccines protect against Sarbecovirus challenge in mice. Science 373:991–998. doi:10.1126/science.abi4506 34214046 PMC8899822

[38] Tan S, Zhao J, Hu X, Li Y, Wu Z, Lu G, Yu Z, Du B, Liu Y, Li L, et al.. 2023. Preclinical evaluation of RQ3013, a broad-spectrum mRNA vaccine against SARS-CoV-2 variants. Sci Bull Sci Found Philipp 68:3192–3206. doi:10.1016/j.scib.2023.11.024 37993332

[39] Li Y, Liu P, Hao T, Liu S, Wang X, Xie Y, Xu K, Lei W, Zhang C, Han P, Li Y, Jin X, Huan Y, Lu Y, Zhang R, Li X, Zhao X, Xu K, Liao P, Lu X, Bi Y, Song H, Wu G, Zhu B, Gao GF. 2023. Rational design of an influenza-COVID-19 chimeric protective vaccine with HA-stalk and S-RBD. Emerg Microbes Infect 12:2231573. doi:10.1080/22221751.2023.2231573 37394992 PMC10348046

[40] Zhao L, Toriumi H, Wang H, Kuang Y, Guo X, Morimoto K, Fu ZF. 2010. Expression of MIP-1alpha (CCL3) by a recombinant rabies virus enhances its immunogenicity by inducing innate immunity and recruiting dendritic cells and B cells. J Virol 84:9642–9648. doi:10.1128/JVI.00326-10 20592092 PMC2937656

[41] Song J, Wang M, Zhou L, Tian P, Sun Z, Sun J, Wang X, Zhuang G, Jiang D, Wu Y, Zhang G. 2023. A candidate nanoparticle vaccine comprised of multiple epitopes of the African swine fever virus elicits a robust immune response. J Nanobiotechnol 21:424. doi:10.1186/s12951-023-02210-9 PMC10647103 37964304

[42] Wan J, Wang C, Wang Z, Wang L, Wang H, Zhou M, Fu ZF, Zhao L. 2024. CXCL13 promotes broad immune responses induced by circular RNA vaccines. Proc Natl Acad Sci USA 121:e2406434121. doi:10.1073/pnas.2406434121 39436660 PMC11536096

[43] Zhou M, Wang L, Zhou S, Wang Z, Ruan J, Tang L, Jia Z, Cui M, Zhao L, Fu ZF. 2015. Recombinant rabies virus expressing dog GM-CSF is an efficacious oral rabies vaccine for dogs. Oncotarget 6:38504–38516. doi:10.18632/oncotarget.5904 26436700 PMC4770717

[44] Wang Z, Li M, Zhou M, Zhang Y, Yang J, Cao Y, Wang K, Cui M, Chen H, Fu ZF, Zhao L. 2017. A novel rabies vaccine expressing CXCL13 enhances humoral immunity by recruiting both T follicular helper and germinal center B cells. J Virol 91:e01956-16. doi:10.1128/JVI.01956-16 27852854 PMC5244327

[45] Ngwuta JO, Chen M, Modjarrad K, Joyce MG, Kanekiyo M, Kumar A, Yassine HM, Moin SM, Killikelly AM, Chuang G-Y, Druz A, Georgiev IS, Rundlet EJ, Sastry M, Stewart-Jones GBE, Yang Y, Zhang B, Nason MC, Capella C, Peeples ME, Ledgerwood JE, McLellan JS, Kwong PD, Graham BS. 2015. Prefusion F-specific antibodies determine the magnitude of RSV neutralizing activity in human sera. Sci Transl Med 7:309ra162. doi:10.1126/scitranslmed.aac4241 PMC4672383 26468324

[46] Che Y, Gribenko AV, Song X, Handke LD, Efferen KS, Tompkins K, Kodali S, Nunez L, Prasad AK, Phelan LM, Ammirati M, Yu X, Lees JA, Chen W, Martinez L, Roopchand V, Han S, Qiu X, DeVincenzo JP, Jansen KU, Dormitzer PR, Swanson KA. 2023. Rational design of a highly immunogenic prefusion-stabilized F glycoprotein antigen for a respiratory syncytial virus vaccine. Sci Transl Med 15:eade6422. doi:10.1126/scitranslmed.ade6422 37023209

[47] Kang YF, Sun C, Sun J, Xie C, Zhuang Z, Xu HQ, Liu Z, Liu YH, Peng S, Yuan RY, Zhao JC, Zeng MS. 2022. Quadrivalent mosaic HexaPro-bearing nanoparticle vaccine protects against infection of SARS-CoV-2 variants. Nat Commun 13:2674. doi:10.1038/s41467-022-30222-w 35562337 PMC9106700

[48] Malebo K, Woodward J, Ximba P, Mkhize Q, Cingo S, Moyo-Gwete T, Moore PL, Williamson AL, Chapman R. 2024. Development of a two-component nanoparticle vaccine displaying an HIV-1 Envelope glycoprotein that elicits tier 2 neutralising antibodies. Vaccines (Basel) 12:1063. doi:10.3390/vaccines12091063 39340093 PMC11436023

[49] Brunette N, Weidle C, Wrenn SP, Fiala B, Ravichandran R, Carr KD, Zak SE, Zumbrun EE, Bakken RR, Murphy M, Chan S, Skotheim R, Borst AJ, Carter L, Correnti CE, Dye JM, Baker D, King NP, Stewart LJ. 2025. A multivalent pan-ebolavirus nanoparticle vaccine provides protection in rodents from lethal infection by adapted Zaire and Sudan viruses. bioRxiv. doi:10.1101/2025.01.29.635581 PMC13547272 42702625

[50] FDA. 2024. Imbalance in severe respiratory syncytial virus (RSV) cases in a clinical trial of an RSV vaccine in infants and young children. Vaccines and related biological products advisory committee meeting (VRBPAC). Available from: https://www.fda.gov/media/184380/download

[51] Olawade DB, Teke J, Fapohunda O, Weerasinghe K, Usman SO, Ige AO, Clement David-Olawade A. 2024. Leveraging artificial intelligence in vaccine development: a narrative review. J Microbiol Methods 224:106998. doi:10.1016/j.mimet.2024.106998 39019262

[52] Feng ZY, Pang XL, Xu Q, Gu ZJ, Li SL, Zhu LL, Li HL. 2025. Design of a multi-valent SARS-CoV-2 peptide vaccine for broad immune protection via deep learning. Engineering (Beijing). doi:10.1016/j.eng.2025.04.025

[53] Shao S, Zhang XF, Hou JW, Yang SS, Han ZB, Wu HL, Tang F, Li XY, Lei ZH, Zhao ZX, Li SX, Liu ZM, Shan P, Jin YQ, Su JG, Liang Y, Zhang J, Li QM. 2024. Design of hepadnavirus core protein-based chimeric virus-like particles carrying epitopes from respiratory syncytial virus. NPJ Vaccines 9:62. doi:10.1038/s41541-024-00855-7 38503757 PMC10951352

[54] Wu X, Li W, Rong H, Pan J, Zhang X, Hu Q, Shi ZL, Zhang XE, Cui Z. 2024. A nanoparticle vaccine displaying conserved epitopes of the preexisting neutralizing antibody confers broad protection against SARS-CoV-2 variants. ACS Nano 18:17749–17763. doi:10.1021/acsnano.4c03075 38935412

[55] Wang Y, Yan A, Song D, Duan M, Dong C, Chen J, Jiang Z, Gao Y, Rao M, Feng J, et al.. 2024. Identification of a highly conserved neutralizing epitope within the RBD region of diverse SARS-CoV-2 variants. Nat Commun 15:842. doi:10.1038/s41467-024-45050-3 38287016 PMC10825162

[56] Sun Y, Liu L, Qiang H, Sun H, Jiang Y, Ren L, Jiang Z, Lei S, Chen L, Wang Y, Lin X, Wang G, Huang Y, Fu Y, Shi Y, Chen X, Yu H, Li S, Luo W, Liu E, Zheng Q, Zheng Z, Xia N. 2024. A potent broad-spectrum neutralizing antibody targeting a conserved region of the prefusion RSV F protein. Nat Commun 15:10085. doi:10.1038/s41467-024-54384-x 39572535 PMC11582626

[57] Huang Y, Song F, Zeng Y, Sun H, Sheng R, Wang X, Liu L, Luo G, Jiang Y, Chen Y, Zhang M, Zhang S, Gu Y, Yu H, Li S, Li T, Zheng Q, Ge S, Zhang J, Xia N. 2025. A single residue switch mediates the broad neutralization of Rotaviruses. Nat Commun 16:838. doi:10.1038/s41467-025-56114-3 39833145 PMC11746992

[58] Ragotte RJ, Tortorici MA, Catanzaro NJ, Addetia A, Coventry B, Froggatt HM, Lee J, Stewart C, Brown JT, Goreshnik I, Sims JN, Milles LF, Wicky BIM, Glögl M, Gerben S, Kang A, Bera AK, Sharkey W, Schäfer A, Harkema JR, Baric RS, Baker D, Veesler D. 2025. Designed miniproteins potently inhibit and protect against MERS-CoV. Cell Rep 44:115760. doi:10.1016/j.celrep.2025.115760 40450691 PMC12276895

[59] Wagh K, Hahn BH, Korber B. 2020. Hitting the sweet spot: exploiting HIV-1 glycan shield for induction of broadly neutralizing antibodies. Curr Opin HIV AIDS 15:267–274. doi:10.1097/COH.0000000000000639 32675574 PMC7877895

[60] Klasse PJ, Sanders RW, Ward AB, Wilson IA, Moore JP. 2025. The HIV-1 envelope glycoprotein: structure, function and interactions with neutralizing antibodies. Nat Rev Microbiol. doi:10.1038/s41579-025-01206-6 40702326

[61] Townsend MJ, Monroe JG, Chan AC. 2010. B-cell targeted therapies in human autoimmune diseases: an updated perspective. Immunol Rev 237:264–283. doi:10.1111/j.1600-065X.2010.00945.x 20727041

[62] Manolova V, Flace A, Bauer M, Schwarz K, Saudan P, Bachmann MF. 2008. Nanoparticles target distinct dendritic cell populations according to their size. Eur J Immunol 38:1404–1413. doi:10.1002/eji.200737984 18389478

[63] Zhao Q, Potter CS, Carragher B, Lander G, Sworen J, Towne V, Abraham D, Duncan P, Washabaugh MW, Sitrin RD. 2014. Characterization of virus-like particles in GARDASIL by cryo transmission electron microscopy. Hum Vaccin Immunother 10:734–739. doi:10.4161/hv.27316 24299977 PMC4130261

[64] Liu ZH, Deng ZF, Lu Y, Fang WH, He F. 2022. A modular and self-adjuvanted multivalent vaccine platform based on porcine circovirus virus-like nanoparticles. J Nanobiotechnol 20:493. doi:10.1186/s12951-022-01710-4 PMC9685936 36424615

[65] Xiao Y, Zhang S, Yan H, Geng X, Wang Y, Xu X, Wang M, Zhang H, Huang B, Pang W, Yang M, Tian K. 2021. The high immunity induced by the virus-like particles of foot-and-mouth disease virus serotype O. Front Vet Sci 8:633706. doi:10.3389/fvets.2021.633706 33718472 PMC7947224

[66] Pliasas VC, Menne Z, Aida V, Yin JH, Naskou MC, Neasham PJ, North JF, Wilson D, Horzmann KA, Jacob J, Skountzou I, Kyriakis CS. 2022. A novel neuraminidase virus-like particle vaccine offers protection against heterologous H3N2 influenza virus infection in the porcine model. Front Immunol 13:915364. doi:10.3389/fimmu.2022.915364 35874791 PMC9300842

[67] Ma J, Xiao X, Zhou Y, Huang W, Sun J, Chang X, Xiao S, Fang L. 2025. Engineering a bivalent nanoparticle vaccine with PCV2 capsid protein and PRRSV epitopes. J Nanobiotechnol 23:438. doi:10.1186/s12951-025-03514-8 PMC12164076 40506710

[68] Li Q, Ma X, Shen Y, Dai J, Nian X, Shang X, Chen J, Wubshet AK, Zhang J, Zheng H. 2024. Chimeric porcine parvovirus VP2 virus-like particles with epitopes of South African serotype 2 foot-and-mouth disease virus elicits specific humoral and cellular responses in mice. Viruses 16:621. doi:10.3390/v16040621 38675963 PMC11054767

[69] Liu X, Zhao T, Wang L, Yang Z, Luo C, Li M, Luo H, Sun C, Yan H, Shu Y. 2023. A mosaic influenza virus-like particles vaccine provides broad humoral and cellular immune responses against influenza A viruses. NPJ Vaccines 8:132. doi:10.1038/s41541-023-00728-5 37679361 PMC10485063

[70] Halfmann PJ, Loeffler K, Duffy A, Kuroda M, Yang JE, Wright ER, Kawaoka Y, Kane RS. 2024. Broad protection against clade 1 sarbecoviruses after a single immunization with cocktail spike-protein-nanoparticle vaccine. Nat Commun 15:1284. doi:10.1038/s41467-024-45495-6 38346966 PMC10861510

[71] Houser KV, Chen GL, Carter C, Crank MC, Nguyen TA, Burgos Florez MC, Berkowitz NM, Mendoza F, Hendel CS, Gordon IJ, et al.. 2022. Safety and immunogenicity of a ferritin nanoparticle H2 influenza vaccine in healthy adults: a phase 1 trial. Nat Med 28:383–391. doi:10.1038/s41591-021-01660-8 35115706 PMC10588819

[72] Widge AT, Hofstetter AR, Houser KV, Awan SF, Chen GL, Burgos Florez MC, Berkowitz NM, Mendoza F, Hendel CS, Holman LA, et al.. 2023. An influenza hemagglutinin stem nanoparticle vaccine induces cross-group 1 neutralizing antibodies in healthy adults. Sci Transl Med 15:eade4790. doi:10.1126/scitranslmed.ade4790 37075129 PMC10619166

[73] Zhang X, Wu S, Liu J, Chen R, Zhang Y, Lin Y, Xi Z, Deng J, Pu Z, Liang C, Feng J, Li R, Lin K, Zhou M, Liu Y, Zhang X, Liu B, Zhang Y, He X, Zhang H. 2023. A mosaic nanoparticle vaccine elicits potent mucosal immune response with significant cross-protection activity against multiple SARS-CoV-2 sublineages. Adv Sci (Weinh) 10:e2301034. doi:10.1002/advs.202301034 37526323 PMC10520630

[74] Bruun TUJ, Andersson A-M, Draper SJ, Howarth M. 2018. Engineering a rugged nanoscaffold to enhance plug-and-display vaccination. ACS Nano 12:8855–8866. doi:10.1021/acsnano.8b02805 30028591 PMC6158681

[75] Cohen AA, van Doremalen N, Greaney AJ, Andersen H, Sharma A, Starr TN, Keeffe JR, Fan C, Schulz JE, Gnanapragasam PNP, Kakutani LM, West AP, Saturday G, Lee YE, Gao H, Jette CA, Lewis MG, Tan TK, Townsend AR, Bloom JD, Munster VJ, Bjorkman PJ. 2022. Mosaic RBD nanoparticles protect against challenge by diverse sarbecoviruses in animal models. Science 377:eabq0839. doi:10.1126/science.abq0839 35857620 PMC9273039

[76] Cohen AA, Keeffe JR, Schiepers A, Dross SE, Greaney AJ, Rorick AV, Gao H, Gnanapragasam PNP, Fan C, West AP, et al.. 2024. Mosaic sarbecovirus nanoparticles elicit cross-reactive responses in pre-vaccinated animals. Cell 187:5554–5571. doi:10.1016/j.cell.2024.07.052 39197450 PMC11460329

[77] Song JY, Choi WS, Heo JY, Lee JS, Jung DS, Kim SW, Park KH, Eom JS, Jeong SJ, Lee J, et al.. 2022. Safety and immunogenicity of a SARS-CoV-2 recombinant protein nanoparticle vaccine (GBP510) adjuvanted with AS03: A randomised, placebo-controlled, observer-blinded phase 1/2 trial. eClinicalMedicine 51:101569. doi:10.1016/j.eclinm.2022.101569 35879941 PMC9304916

[78] Richner JM, Himansu S, Dowd KA, Butler SL, Salazar V, Fox JM, Julander JG, Tang WW, Shresta S, Pierson TC, Ciaramella G, Diamond MS. 2017. Modified mRNA vaccines protect against Zika virus infection. Cell 168:1114–1125. doi:10.1016/j.cell.2017.02.017 28222903 PMC5388441

[79] Liu J, Sun J, Ding X, Liu W, Wang Y, Wang Z, Peng H, Zhang Y, Su W, Jiang C. 2024. A nucleoside-modified mRNA vaccine forming rabies virus-like particle elicits strong cellular and humoral immune responses against rabies virus infection in mice. Emerg Microbes Infect 13:2389115. doi:10.1080/22221751.2024.2389115 39129566 PMC11328811

[80] Hendricks GG, Grigoryan L, Navarro MJ, Catanzaro NJ, Hubbard ML, Powers JM, Mattocks M, Treichel C, Walls AC, Lee J, et al.. 2024. Computationally designed mRNA-launched protein nanoparticle vaccines. bioRxiv:2024.07.22.604655. doi:10.1101/2024.07.22.604655 PMC13035345 41091918

[81] Zhang P, Narayanan E, Liu Q, Tsybovsky Y, Boswell K, Ding S, Hu Z, Follmann D, Lin Y, Miao H, et al.. 2021. A multiclade env-gag VLP mRNA vaccine elicits tier-2 HIV-1-neutralizing antibodies and reduces the risk of heterologous SHIV infection in macaques. Nat Med 27:2234–2245. doi:10.1038/s41591-021-01574-5 34887575

[82] Zhang P, Falcone S, Tsybovsky Y, Singh M, Gopan V, Miao H, Seo Y, Rogers D, Renzi I, Lai YT, Narayanan E, Stewart-Jones G, Himansu S, Carfi A, Fauci AS, Lusso P. 2023. Increased neutralization potency and breadth elicited by a SARS-CoV-2 mRNA vaccine forming virus-like particles. Proc Natl Acad Sci USA 120:e2305896120. doi:10.1073/pnas.2305896120 37428933 PMC10629519

[83] Hoffmann MAG, Yang Z, Huey-Tubman KE, Cohen AA, Gnanapragasam PNP, Nakatomi LM, Storm KN, Moon WJ, Lin PJC, West AP Jr, Bjorkman PJ. 2023. ESCRT recruitment to SARS-CoV-2 spike induces virus-like particles that improve mRNA vaccines. Cell 186:2380–2391. doi:10.1016/j.cell.2023.04.024 37146611 PMC10121106

[84] Yin D, Zhong Y, Ling S, Lu S, Wang X, Jiang Z, Wang J, Dai Y, Tian X, Huang Q, et al.. 2025. Dendritic-cell-targeting virus-like particles as potent mRNA vaccine carriers. Nat Biomed Eng 9:185–200. doi:10.1038/s41551-024-01208-4 38714892

[85] Mohsen MO, Zha L, Cabral-Miranda G, Bachmann MF. 2017. Major findings and recent advances in virus-like particle (VLP)-based vaccines. Semin Immunol 34:123–132. doi:10.1016/j.smim.2017.08.014 28887001

[86] Link A, Zabel F, Schnetzler Y, Titz A, Brombacher F, Bachmann MF. 2012. Innate immunity mediates follicular transport of particulate but not soluble protein antigen. J Immunol 188:3724–3733. doi:10.4049/jimmunol.1103312 22427639

[87] Hadj Hassine I, Ben M’hadheb M, Almalki MA, Gharbi J. 2024. Virus‐like particles as powerful vaccination strategy against human viruses. Rev Med Virol 34:e2498. doi:10.1002/rmv.2498 38116958

[88] Bachmann MF, van Damme P, Lienert F, Schwarz TF. 2025. Virus-like particles: a versatile and effective vaccine platform. Expert Rev Vaccines 24:444–456. doi:10.1080/14760584.2025.2508517 40387310

[89] Laliberté-Gagné ME, Bolduc M, Garneau C, Olivera-Ugarte SM, Savard P, Leclerc D. 2021. Modulation of antigen display on PapMV nanoparticles influences its immunogenicity. Vaccines (Basel) 9:33. doi:10.3390/vaccines9010033 33435570 PMC7829862

[90] Kang S, Uchida M, O’Neil A, Li R, Prevelige PE, Douglas T. 2010. Implementation of p22 viral capsids as nanoplatforms. Biomacromolecules 11:2804–2809. doi:10.1021/bm100877q 20839852

[91] Wang G, Liu Y, Feng H, Chen Y, Yang S, Wei Q, Wang J, Liu D, Zhang G. 2018. Immunogenicity evaluation of MS2 phage-mediated chimeric nanoparticle displaying an immunodominant B cell epitope of foot-and-mouth disease virus. PeerJ 6:e4823. doi:10.7717/peerj.4823 29844975 PMC5970553

[92] Liu X, Chang X, Rothen D, Derveni M, Krenger P, Roongta S, Wright E, Vogel M, Tars K, Mohsen MO, Bachmann MF. 2021. AP205 VLPs based on dimerized capsid proteins accommodate RBM domain of SARS-CoV-2 and serve as an attractive vaccine candidate. Vaccines (Basel) 9:403. doi:10.3390/vaccines9040403 33921677 PMC8073683

[93] Skamel C, Aller SG, Bopda Waffo A. 2018. Correction: In vitro evolution and affinity-maturation with coliphage Qβ display. PLoS ONE 13:e0199953. doi:10.1371/journal.pone.0199953 29944705 PMC6019667

[94] Karan S, Affonso De Oliveira JF, Moreno-Gonzalez MA, Steinmetz NF. 2024. A self-amplifying human papillomavirus 16 vaccine candidate delivered by tobacco Mosaic Virus-Like Particles. ACS Appl Bio Mater 7:7675–7683. doi:10.1021/acsabm.4c01239 PMC11648571 39512153

[95] Huang X, Wang X, Zhang J, Xia N, Zhao Q. 2017. Escherichia coli-derived virus-like particles in vaccine development. NPJ Vaccines 2:3. doi:10.1038/s41541-017-0006-8 29263864 PMC5627247

[96] Kheirvari M, Liu H, Tumban E. 2023. Virus-like particle vaccines and platforms for vaccine development. Viruses 15:1109. doi:10.3390/v15051109 37243195 PMC10223759

[97] Zhang X, Meining W, Fischer M, Bacher A, Ladenstein R. 2001. X-ray structure analysis and crystallographic refinement of lumazine synthase from the hyperthermophile Aquifex aeolicus at 1.6 A resolution: determinants of thermostability revealed from structural comparisons. J Mol Biol 306:1099–1114. doi:10.1006/jmbi.2000.4435 11237620

[98] Roier S, Mangala Prasad V, McNeal MM, Lee KK, Petsch B, Rauch S. 2023. mRNA-based VP8 nanoparticle vaccines against rotavirus are highly immunogenic in rodents. NPJ Vaccines 8:190. doi:10.1038/s41541-023-00790-z 38129390 PMC10739717

[99] He L, de Val N, Morris CD, Vora N, Thinnes TC, Kong L, Azadnia P, Sok D, Zhou B, Burton DR, Wilson IA, Nemazee D, Ward AB, Zhu J. 2016. Presenting native-like trimeric HIV-1 antigens with self-assembling nanoparticles. Nat Commun 7:12041. doi:10.1038/ncomms12041 27349934 PMC4931238

[100] Bale JB, Gonen S, Liu Y, Sheffler W, Ellis D, Thomas C, Cascio D, Yeates TO, Gonen T, King NP, Baker D. 2016. Accurate design of megadalton-scale two-component icosahedral protein complexes. Science 353:389–394. doi:10.1126/science.aaf8818 27463675 PMC5485857

[101] Leekha A, Saeedi A, Sefat KMSR, Kumar M, Martinez-Paniagua M, Damian A, Kulkarni R, Reichel K, Rezvan A, Masoumi S, Liu X, Cooper LJN, Sebastian M, Sands CM, Das VE, Patel NB, Hurst B, Varadarajan N. 2025. Multi-antigen intranasal vaccine protects against challenge with sarbecoviruses and prevents transmission in hamsters. Nat Commun 15:6193. doi:10.1038/s41467-024-50133-2 PMC11266618 39043645

[102] Fan C, Keeffe JR, Malecek KE, Cohen AA, West AP, Baharani VA, Rorick AV, Gao H, Gnanapragasam PNP, Rho S, Alvarez J, Segovia LN, Hatziioannou T, Bieniasz PD, Bjorkman PJ. 2025. Cross-reactive sarbecovirus antibodies induced by mosaic RBD-nanoparticles. bioRxiv:2025.01.02.631145. doi:10.1101/2025.01.02.631145 PMC12130868 40402246

[103] Liu C, Xu S, Zheng Y, Xie Y, Xu K, Chai Y, Luo T, Dai L, Gao GF. 2024. Mosaic RBD nanoparticle elicits immunodominant antibody responses across sarbecoviruses. Cell Rep 43:114235. doi:10.1016/j.celrep.2024.114235 38748880

[104] Hills RA, Tan TK, Cohen AA, Keeffe JR, Keeble AH, Gnanapragasam PNP, Storm KN, Rorick AV, West AP, Hill ML, Liu S, Gilbert-Jaramillo J, Afzal M, Napier A, Admans G, James WS, Bjorkman PJ, Townsend AR, Howarth MR. 2024. Proactive vaccination using multiviral Quartet Nanocages to elicit broad anti-coronavirus responses. Nat Nanotechnol 19:1216–1223. doi:10.1038/s41565-024-01655-9 38710880 PMC11329374

[105] Kim J, Eygeris Y, Gupta M, Sahay G. 2021. Self-assembled mRNA vaccines. Adv Drug Deliv Rev 170:83–112. doi:10.1016/j.addr.2020.12.014 33400957 PMC7837307

[106] Manzanares D, Ceña V. 2020. Endocytosis: the nanoparticle and submicron nanocompounds gateway into the cell. Pharmaceutics 12:371. doi:10.3390/pharmaceutics12040371 32316537 PMC7238190

[107] Chatterjee S, Kon E, Sharma P, Peer D. 2024. Endosomal escape: a bottleneck for LNP-mediated therapeutics. Proc Natl Acad Sci USA 121:e2307800120. doi:10.1073/pnas.2307800120 38437552 PMC10945858

[108] Votteler J, Sundquist WI. 2013. Virus budding and the ESCRT pathway. Cell Host Microbe 14:232–241. doi:10.1016/j.chom.2013.08.012 24034610 PMC3819203

[109] Ling S, Yang S, Hu X, Yin D, Dai Y, Qian X, Wang D, Pan X, Hong J, Sun X, Yang H, Paludan SR, Cai Y. 2021. Lentiviral delivery of co-packaged Cas9 mRNA and a VEGFA-targeting guide RNA prevents wet age-related macular degeneration in mice. Nat Biomed Eng 5:144–156. doi:10.1038/s41551-020-00656-y 33398131

[110] Kim J, Eygeris Y, Ryals RC, Jozić A, Sahay G. 2024. Strategies for non-viral vectors targeting organs beyond the liver. Nat Nanotechnol 19:428–447. doi:10.1038/s41565-023-01563-4 38151642

[111] Witten J, Hu Y, Langer R, Anderson DG. 2024. Recent advances in nanoparticulate RNA delivery systems. Proc Natl Acad Sci USA 121:e2307798120. doi:10.1073/pnas.2307798120 38437569 PMC10945842

[112] Kheirolomoom A, Kare AJ, Ingham ES, Paulmurugan R, Robinson ER, Baikoghli M, Inayathullah M, Seo JW, Wang J, Fite BZ, Wu B, Tumbale SK, Raie MN, Cheng RH, Nichols L, Borowsky AD, Ferrara KW. 2022. In situ T-cell transfection by anti-CD3-conjugated lipid nanoparticles leads to T-cell activation, migration, and phenotypic shift. Biomaterials 281:121339. doi:10.1016/j.biomaterials.2021.121339 35078042 PMC8892572

[113] Shi D, Toyonaga S, Anderson DG. 2023. In vivo RNA delivery to hematopoietic stem and progenitor cells via targeted lipid nanoparticles. Nano Lett 23:2938–2944. doi:10.1021/acs.nanolett.3c00304 36988645 PMC10103292

[114] Goldinger SM, Dummer R, Baumgaertner P, Mihic-Probst D, Schwarz K, Hammann-Haenni A, Willers J, Geldhof C, Prior JO, Kündig TM, Michielin O, Bachmann MF, Speiser DE. 2016. Nano-particle vaccination combined with TLR-7 and -9 ligands triggers memory and effector CD8^+^ T-cell responses in melanoma patients. Eur J Immunol 46:493. 10.1002/eji.201142361 PMC3549564 22806397

[115] Dowling DJ, Scott EA, Scheid A, Bergelson I, Joshi S, Pietrasanta C, Brightman S, Sanchez-Schmitz G, Van Haren SD, Ninković J, Kats D, Guiducci C, de Titta A, Bonner DK, Hirosue S, Swartz MA, Hubbell JA, Levy O. 2017. Toll-like receptor 8 agonist nanoparticles mimic immunomodulating effects of the live BCG vaccine and enhance neonatal innate and adaptive immune responses. J Allergy Clin Immunol 140:1339–1350. doi:10.1016/j.jaci.2016.12.985 28343701 PMC5667586

[116] Yong H, Tian Y, Li Z, Wang C, Zhou D, Liu J, Huang X, Li J. 2025. Highly branched poly(β-amino ester)s for efficient mRNA delivery and nebulization treatment of silicosis. Adv Mater 37:e2414991. doi:10.1002/adma.202414991 40167376

[117] Zhang H, Leal J, Soto MR, Smyth HDC, Ghosh D. 2020. Aerosolizable lipid nanoparticles for pulmonary delivery of mRNA through design of experiments. Pharmaceutics 12:1042. doi:10.3390/pharmaceutics12111042 33143328 PMC7692784

[118] Wang J, Zhang Y, Jia Y, Xing H, Xu F, Xia B, Lai W, Yuan Y, Li X, Shan S, Chen J, Guo W, Zhang J, Zheng A, Li J, Gong N, Liang XJ. 2025. Targeting vaccines to dendritic cells by mimicking the processing and presentation of antigens in xenotransplant rejection. Nat Biomed Eng 9:201–214. doi:10.1038/s41551-025-01343-6 39948171

[119] Srivastava P, Rütter M, Antoniraj GM, Ventura Y, David A. 2024. Dendritic cell-targeted nanoparticles enhance T cell activation and antitumor immune responses by boosting antigen presentation and blocking PD-L1 pathways. ACS Appl Mater Interfaces 16:53577–53590. doi:10.1021/acsami.4c12821 39344665

[120] Gou S, Liu W, Wang S, Chen G, Chen Z, Qiu L, Zhou X, Wu Y, Qi Y, Gao Y. 2021. Engineered nanovaccine targeting Clec9a^+^ dendritic cells remarkably enhances the cancer immunotherapy effects of STING agonist. Nano Lett 21:9939–9950. doi:10.1021/acs.nanolett.1c03243 34779631

[121] Liu J, Cui Y, Cabral H, Tong A, Yue Q, Zhao L, Sun X, Mi P. 2024. Glucosylated nanovaccines for dendritic cell-targeted antigen delivery and amplified cancer immunotherapy. ACS Nano 18:25826–25840. doi:10.1021/acsnano.4c09053 39196858

[122] Zhuang X, Qi Y, Wang M, Yu N, Nan F, Zhang H, Tian M, Li C, Lu H, Jin N. 2020. mRNA vaccines encoding the HA protein of influenza A H1N1 virus delivered by cationic lipid nanoparticles induce protective immune responses in mice. Vaccines (Basel) 8:123. doi:10.3390/vaccines8010123 32164372 PMC7157730

[123] Wan J, Wang Z, Wang L, Wu L, Zhang C, Zhou M, Fu ZF, Zhao L. 2024. Circular RNA vaccines with long-term lymph node-targeting delivery stability after lyophilization induce potent and persistent immune responses. mBio 15:e0177523. doi:10.1128/mbio.01775-23 38078742 PMC10790773

[124] Grødeland G, Fossum E, Bogen B. 2015. Polarizing T and B cell responses by APC-targeted subunit vaccines. Front Immunol 6:367. doi:10.3389/fimmu.2015.00367 26257735 PMC4507452

[125] Madan RA, Gulley JL. 2011. Sipuleucel-T: harbinger of a new age of therapeutics for prostate cancer. Expert Rev Vaccines 10:141–150. doi:10.1586/erv.10.173 21332262 PMC3460263

[126] van der Zande HJP, Nitsche D, Schlautmann L, Guigas B, Burgdorf S. 2021. The mannose receptor: from endocytic receptor and biomarker to regulator of (Meta) inflammation. Front Immunol 12:765034. doi:10.3389/fimmu.2021.765034 34721436 PMC8551360

[127] Wang Z, Hood ED, Nong J, Ding J, Marcos‐Contreras OA, Glassman PM, Rubey KM, Zaleski M, Espy CL, Gullipali D, Miwa T, Muzykantov VR, Song W, Myerson JW, Brenner JS. 2022. Combating complement's deleterious effects on nanomedicine by conjugating complement regulatory proteins to nanoparticles. Adv Mater Weinheim 34:e2107070. doi:10.1002/adma.202107070 PMC9062787 34910334

[128] Suk JS, Xu Q, Kim N, Hanes J, Ensign LM. 2016. PEGylation as a strategy for improving nanoparticle-based drug and gene delivery. Adv Drug Deliv Rev 99:28–51. doi:10.1016/j.addr.2015.09.012 26456916 PMC4798869

[129] Estapé Senti M, de Jongh CA, Dijkxhoorn K, Verhoef JJF, Szebeni J, Storm G, Hack CE, Schiffelers RM, Fens MH, Boross P. 2022. Anti-PEG antibodies compromise the integrity of PEGylated lipid-based nanoparticles via complement. J Control Release 341:475–486. doi:10.1016/j.jconrel.2021.11.042 34890719

[130] Zaleski MH, Chase LS, Hood ED, Wang Z, Nong J, Espy CL, Zamora ME, Wu J, Morrell LJ, Muzykantov VR, Myerson JW, Brenner JS. 2025. Conjugation chemistry markedly impacts toxicity and biodistribution of targeted nanoparticles, mediated by complement activation. Adv Mater 37:e2409945. doi:10.1002/adma.202409945 39663706 PMC11795710

[131] Xiao Q, Zoulikha M, Qiu M, Teng C, Lin C, Li X, Sallam MA, Xu Q, He W. 2022. The effects of protein corona on in vivo fate of nanocarriers. Adv Drug Deliv Rev 186:114356. doi:10.1016/j.addr.2022.114356 35595022

[132] van den Born E, Olasz F, Mészáros I, Göltl E, Oláh B, Joshi J, van Kilsdonk E, Segers R, Zádori Z. 2025. African swine fever virus vaccine strain ASFV-G-∆I177l reverts to virulence and negatively affects reproductive performance. NPJ Vaccines 10:46. doi:10.1038/s41541-025-01099-9 40050309 PMC11885574

[133] Zhao S, Gao K, Han H, Stenzel M, Yin B, Song H, Lawanprasert A, Nielsen JE, Sharma R, Arogundade OH, Pimcharoen S, Chen YJ, Paul A, Tuma J, Collins MG, Wyle Y, Cranick MG, Burgstone BW, Perez BS, Barron AE, Smith AM, Lee HY, Wang A, Murthy N. 2024. Acid-degradable lipid nanoparticles enhance the delivery of mRNA. Nat Nanotechnol 19:1702–1711. doi:10.1038/s41565-024-01765-4 39179796 PMC12479011

[134] Meng Y, Yao Z, Ke X, Hu M, Ren H, Gao S, Zhang H. 2025. Extracellular vesicles-based vaccines: Emerging immunotherapies against cancer. J Control Release 378:438–459. doi:10.1016/j.jconrel.2024.12.010 39667569

[135] Fu W, Guo M, Zhou X, Wang Z, Sun J, An Y, Guan T, Hu M, Li J, Chen Z, Ye J, Gao X, Gao GF, Dai L, Wang Y, Chen C. 2024. Injectable hydrogel mucosal vaccine elicits protective immunity against respiratory viruses. ACS Nano 18:11200–11216. doi:10.1021/acsnano.4c00155 38620102

[136] Atalis A, Keenum MC, Pandey B, Beach A, Pradhan P, Vantucci C, O’Farrell L, Noel R, Jain R, Hosten J, Smith C, Kramer L, Jimenez A, Ochoa MA, Frey D, Roy K. 2022. Nanoparticle-delivered TLR4 and RIG-I agonists enhance immune response to SARS-CoV-2 subunit vaccine. J Control Release 347:476–488. doi:10.1016/j.jconrel.2022.05.023 35577151 PMC9121740

